# Microbial allies against drought stress: an optimized screening method to improve seedling survival for forest restorations

**DOI:** 10.1186/s40793-026-00878-8

**Published:** 2026-03-25

**Authors:** Sonja Magosch, Claudia Barrera, Adrian Bölz, Karin Pritsch, Michael Rothballer, J. Philipp Benz

**Affiliations:** 1 TUM School of Life Sciences, Professorship for Fungal Biotechnology in Wood Science, Technical University of Munich, Freising, Germany; 2 TUM School of Life Sciences, Professorship for Land Surface-Atmosphere Interactions, Technical University of Munich, Freising, Germany; 3 Research Unit Environmental Simulation, Helmholtz Munich, Neuherberg, Germany; 4 Institute of Network Biology, Helmholtz Munich, Neuherberg, Germany

**Keywords:** Drought tolerance, Tree seedlings, Plant growth-promoting microorganisms, Reforestation, In vivo plant screening

## Abstract

**Background:**

Improving drought tolerance of tree seedlings by plant growth-promoting microorganisms (PGPMs) is a promising approach for nature-based forest restoration. Identifying suitable microorganisms requires a robust selection, including efficient *in planta* screenings.

**Results:**

We sampled at two forest sites in southern Germany with drought legacies and within a dry period to enhance the probability of isolating drought-tolerant microbes. Metabarcoding of the resident soil community revealed a broad on-site diversity with the potential for diverse plant growth-promoting and stress-resistance traits. We isolated 1,292 bacteria and 59 fungi from fine roots of Norway spruce and European beech. 429 isolates were identified to the Genus level. The most abundant genera were *Paraburkholderia* (121) and *Bacillus* (43) in bacteria and *Penicillium* (8) and *Umbelopsis* (8) in fungi. Isolates were scored in vitro for abiotic stress tolerance and plant growth-promoting traits, revealing diverse plant growth-promoting abilities for 31 bacteria and a particularly high stress tolerance for 8 fungi. Importantly, an axenic 24-well plate system was developed to investigate the influence of bacteria on spruce seedlings under drought conditions. The system allowed direct comparison of inoculation effects on seedling growth and survival with or without drought application. Six bacterial strains significantly promoted plant growth under well-watered conditions, while two bacterial strains improved survival and root length under drought.

**Conclusions:**

This study represents one of the first larger scale screenings for PGPMs isolated from forest soils on tree seedlings under drought and may contribute to finding nature-based drought mitigation strategies.

**Supplementary Information:**

The online version contains supplementary material available at 10.1186/s40793-026-00878-8.

## Introduction

In recent decades, droughts have increased in duration and frequency [1–3]. They are characterized by below-average precipitation and high evaporation rates over a prolonged period, often accompanied by increased temperatures [4]. In European forests, increased drought leads to elevated tree mortality [5–7], often resulting in failed regeneration or shifts in tree species composition [8]. Forests cover one third of the terrestrial area and are important players in climate regulation and protection, for example through nutrient cycling and storage of large amounts of anthropogenic and atmospheric carbon [9–11]. As a result, shifts or degradation of forests become major issues for global carbon fluxes.

At the plant level, drought leads to restricted soil nutrient assimilation as well as morphological changes, including reduced height and size of leaves, stems, shoots and roots [12, 13]. This is particularly relevant in seedlings [14, 15]. Due to their shallow root system and growth in the upper soil layers, they are more susceptible to desiccation by evapotranspiration. Furthermore, unlike mature trees, seedlings do not have sufficient carbon reserves to be used during periods of stress to maintain physiological functions [16]. To prevent water loss, stomata are closed early, leading to a lower O_2_-/CO_2_-exchange, carbon starvation [8, 17] and ultimately to the accumulation of harmful reactive oxygen species [18, 19]. In addition, endogenous phytohormone levels such as auxin or abscisic acid, which are involved in plant stress defense and plant growth regulation, are misregulated under drought [20, 21].

Forests in Central Europe are currently dominated by Norway spruce (*Picea abies*) and European beech (*Fagus sylvatica*), making up 30% of the forest area, and being of high importance for the provision of timber [22–24]. Compared to beech, spruce is even more susceptible to abiotic and biotic disturbances such as limited water supply, storms, and pathogen attacks, among other things due to its superficial root system and an associated reduced competitiveness [1, 22, 25].

Forest restoration relies on the successful establishment of seedlings, which is particularly challenging under drought [14, 26, 27]. Different measures have been tested to mitigate the negative impacts of drought on seedling survival, growth, and stress resilience. One option is the use of chemical fertilizers as they support faster growth [28, 29]. However, this strategy is associated with high costs, can negatively affect the environment, and contributes to the depletion of non-renewable resources [30, 31]. Recently, other, more sustainable options have gained importance. The application of biofertilizers comprising plant-beneficial bacteria and fungi is a promising approach in agricultural production systems [32], and inoculation with plant growth-promoting microorganisms (PGPMs) is an eco-friendly and financially advantageous alternative to chemical fertilizers [31, 33, 34]. PGPMs are free-living in the rhizosphere soil, rhizoplane, phyllosphere or endosphere, and can promote plant growth or reduce biotic or abiotic stress through a variety of functional traits [18, 35, 36] involving direct and indirect mechanisms [37]. Direct ways comprise promotion of plant growth by influencing phytohormone levels, for example through indole-3-acetic acid (IAA) production or by producing 1-aminocyclopropane-1-carboxylate (ACC) deaminase, hydrolyzing the ethylene precursor ACC. They also include improved nutrient supply via siderophore production, phosphate solubilization or nitrogen fixation. Indirect strategies include pathogen suppression and induced systemic resistance [13, 31, 38–40].

Additionally, tree seedlings inoculated with microbiota that had previously experienced drought stress, showed a higher survival rate under drought [41], making the application of stress-primed microorganisms an important strategy for mitigating the negative effects of drought.

Many plant growth-promoting bacteria (PGPB) and fungi have been characterized for their efficacy in agriculture, and some of those are already commercially utilized, e.g. the bacterial genera *Azospirillum* spp., *Bacillus* spp., *Burkholderia* spp., *Pseudomonas* spp. and *Streptomyces* spp., as well as *Serratia* spp. and *Rhizobium* [18, 31, 32, 36, 39]. Fungi used in agriculture include genera like *Trichoderma* spp., *Aspergillus* spp., or arbuscular mycorrhizal fungi [42, 43]. *P. abies* and *F. sylvatica* form ectomycorrhizae with specific Basidio- and Ascomycota [44]. Their beneficial effects on seedling establishment and drought tolerance are well studied [22, 44–46], but their wider application remains limited due to cultivation challenges and difficulties in field establishment. In our study, we focused instead on more easily culturable PGPMs, including bacteria and root-associated, mainly saprotrophic fungi.

Despite the central importance of tree root microbiomes for nutrient and water acquisition and protection against pathogens [47], there is only very limited information on PGPMs in boreal and temperate forest ecosystems [48, 49]. *Paraburkholderia*, *Serratia* and *Bacillus* spp. displayed plant growth-promoting (PGP) traits in vitro and enhanced seedling germination or growth of different pine species [50, 51]. A plant growth-promoting *Paenibacillus* strain was isolated from a spruce forest in China, but its PGP potential was only demonstrated for clover and *Arabidopsis* [52]. There are some rare examples of PGPB tested on spruce [53, 54], where seedlings were inoculated with the plant-beneficial *Streptomyces* AcH505 strain, which protected the seedlings from *Heterobasidion* *annosum* infection, promoted root branching and slightly increased lateral root formation. Another study identified a *Pseudomonas* strain and two *Bacillus* strains, which had beneficial effects on hybrid spruce growth, and increased seedling dry weight compared to uninoculated controls [55].

However, to date, no studies have investigated the use of PGPMs to improve drought tolerance in spruce seedlings. To support successful seedling establishment, a rapid and targeted screening for a broad range of PGPMs is essential, ideally involving a direct application to the plant of interest.

To overcome these limitations, a screening for plant-beneficial bacterial and fungal isolates was initiated which, in addition to a classical in vitro screening, included an optimized axenic plant cultivation system using 24-deep-well plates with spruce seedlings, being particularly drought-sensitive. To increase the probability of finding species being effective under drought, isolates were extracted after one week of low or no precipitation from soils with moderate-dry to dry climate legacies in southern Germany. Prior to isolation, on-site diversity was analyzed by amplicon sequencing to estimate the potential for obtaining a broad strain collection with PGP and stress resistance properties. By isolation and selection via in vitro assays, promising isolates were filtered out from the total microbial pool.

Isolated strains belonging to genera which already contain well-described PGP strains, particularly in spruce, were preferentially, though not exclusively, chosen for in vitro characterization. On the one hand, we followed the hypothesis that the chances of finding high genetic potential for beneficial associations with plants will be best in relatives to known PGP strains. On the other hand, it is well supported that isolates from the same genus or even species can exhibit additional or optimized functional traits, rendering them more efficient or better adapted to certain environments or hosts [56–58]. Such considerable functional variability within both bacterial and fungal genera underscores the critical importance of characterization at the strain level to identify isolates with the desired traits.

Taken together, this study aimed to develop microbe-based strategies for enhancing drought stress tolerance in spruce seedlings. By integrating field microbiome profiling, functional and single-strain characterization and *in planta* screening, it contributes to developing practical approaches for forest restoration.

## Material and methods

### Study sites and rhizosphere soil sampling

Samples for isolation and amplicon sequencing were collected in summer 2021 from two mixed stands of Norway spruce (*Picea abies*) and European beech (*Fagus sylvatica*) in southern Germany, differing in long-term precipitation and soil properties. The moderately dry site Kranzberger Forst (MAP 854 mm, MAT 8.2 °C [59]), located at 48°25′8.4″N, 11°39′39.6″E, is characterized by loess over tertiary sediments, with a luvisol soil type [22, 59]. Samples were taken from plots previously subjected to drought stress in late July, following precipitation of 0–8.7 mm in the preceding week (Table S1). The drier site Kelheim (MAP 713 mm, MAT 8.5 °C [59]), located at 48°56′8.16″N, 11°49′19.2″E, features loess over weathered limestone with a cambisol soil type [22, 59]. Samples from this site were collected in early September after a week without precipitation (Table S1). Soil pH conditions at the two sites were previously measured by [59] and [60], and ranged between 4.0 and 4.5 in the upper 10 cm of the soil.

For amplicon sequencing with the aim to analyze the microbial community composition at the time of sampling, 5 × 5 soil cores (4.5 cm diameter to 10 cm depth), including root material, were collected at each site from below 3 adjacent beech or spruce trees. Samples were immediately frozen, transported on dry ice and stored at -80 °C until usage. Only fine roots with adhering soil (rhizosphere soil) were used for further processing (Section “Sample preparation for amplicon sequencing to characterize the root-associated microbial communities”).

For bacterial and fungal isolation, rhizosphere soil samples were taken directly below the trees by tracking fine roots (< 1 mm diameter) from coarser roots. Five samples per tree species were taken at each site, each sample consisting of 3–5 fine roots with adhering soil. Samples were kept at 4 °C during transport and processed on the day of sampling to avoid changes in the microbial composition.

### Sample preparation for amplicon sequencing to characterize the root-associated microbial communities

DNA was extracted from rhizosphere soil using the FastDNA Spin kit for soil (MP biomedicals, Hessen, Germany), following manufacturer’s instructions. DNA concentration was measured at a Nanodrop® One spectrometer (Thermo Fisher Scientific, Waltham, MA, USA) and adjusted to 1.2–15 ng µL^−1^. DNA-samples were sent to the Core Facility of Microbiome, ZIEL, TUM (Freising, Weihenstephan; PD Dr. Klaus Neuhaus) for library preparation, purification, and Next Generation Sequencing (Illumina MiSeq® reagent kit v3 (600 cycle), paired-end reads). Nuclease-free water served as a non-template negative control, and ZymoBIOMICS® Microbial Community Standard (Zymo Research Europe, Freiburg im Breisgau, Germany) as positive control. Primers 515F (GTGYCAGCMGCCGCGGTAA, [61]) and 806R (GGACTACNVGGGTWTCTAAT, [62]) were used to sequence the v4 region of the 16S rRNA gene. The first-step PCR amplification for bacteria was performed with an initial denaturation at 98 °C for 40 s, followed by 20 cycles of 98 °C for 20 s, 55 °C for 40 s and 72 °C for 40 s, with a final elongation at 72 °C for 2 min. The second‑step PCR was performed for 12 cycles under the same conditions. The fungal ITS2 region was amplified using a mixture of different primers according to [63] with adaptations: ITS3-Mix123-fw (AHCGATGAAGAACGCAG), ITS3-Mix45-fw (CATCGATGAAGAACGTRG), ITS4-Mix1-rv (TCCTCCGCTTATTGATATGC), ITS4-Mix2-rv (TCCTGCGCTTATTGATATGC), ITS4-Mix3-rv (TCCTCGCCTTATTGATATGC) and ITS4-Mix4-rv (TCCTCCGCTGAWTAATATGC). For fungi, the first‑step PCR amplification conditions were the same as for bacteria, whereas the second‑step PCR was performed for 15 instead of 12 cycles. After demultiplexing, the data was provided from the Core Facility of Microbiome as fastq files. The raw sequencing data have been deposited in the NCBI Sequence Read Archive (SRA) under the following accession numbers: SRR33458690-SRR33458820 for bacteria and SRR33459453-SRR33459578 for fungi (BioProject: PRJNA1259936).

Sequencing data was processed using QIIME2 (v2024.2.0, [64]) with the standard trimming and denoised using DADA2 [65]. Taxonomic classification of Amplicon sequence variants (ASVs) was performed using Greengenes2 (v2022.10, [66]) for the 16S rRNA gene amplicons, while ITS region amplicons were classified using the “feature-classifier” trained on the UNITE database (v10.0 [67],). Samples with less than 8,000 reads, in the case of 16S rRNA gene, or less than 3,000 reads, in the case of fungi, as well as singletons were removed. Overall, 95 samples were retained while 5 samples were removed due to a low sequencing depth. ASVs found in low abundance were removed. Samples were rarefied to the lowest depth using the “Rarefy” function of the *GUniFrac* R packages [68]. Community composition was evaluated using the *microbiome* R packages (v1.24.0, [69]). Bray–Curtis distance was calculated using the R package *vegan* (v2.6.6, [70]), and a Principal Coordinates Analysis (PCoA) was performed to compare similarities between different groups. Functional profiles were predicted using Tax4Fun2 [71] with the default database and a 97% similarity cut-off. The predicted enzymes were then manually inspected to identify those involved in the pathways evaluated in the in vitro assays. Plots were generated using ggplot2 [72] or Origin Pro (version 2021b).

### Bacterial and fungal isolation and cultivation

Rhizosphere soil was grinded in 1 mL 1× phosphate buffered saline (1× PBS) using autoclaved mortar and pestle, diluted up to a factor of 10^–3^, and 100 µL of the suspension was plated on petri dishes on different solid agar media in 10 replicates per medium: King’s B medium (Fluka Biochemika, Germany), M9 minimal medium (after Arie Gerloff, 20% (w/v) glucose), and Modified Melin-Norkrans (MMNC) medium [73]. For *Streptomyces* isolation, 500 µL of ground rhizosphere soil was transferred into Hayakawa and Nonomura liquid medium with CaCl_2_ (HNC; in g L^−1^: yeast extract, 60; SDS, 0.5; CaCl_2_, 0.5; in ddH_2_O, pH 7.0), modified from [74], and incubated at 120 rpm and 42 °C for 30 min. The suspension was filtered through glass wool and diluted up to a factor of 10^–1^ and 10^–2^, and 100 µL of both dilutions were plated on International Streptomyces Project-2 (ISP-2)- and Humic acid-vitamin (HV)-agar [75, 76]. Plates were incubated at 27 °C for 1–2 weeks. For isolation of fungi, the same dilutions as for bacteria were used and 100 µL were plated on solid MMNC medium plates and, for some samples, additionally on King’s B and M9 medium. Two fungal isolates (*Lycoperdon* sp. F31, *Collybiopsis* sp. F32) were isolated by placing small pieces from the inside of the fruiting bodies on MMNC medium. All plates were incubated at 25 °C for up to 10 days. For purification and subculturing, part of the mycelium or spores were transferred to fresh MMNC medium where they were grown for 10 days at 25 °C and checked daily for potential bacterial contamination.

Bacteria were pre-cultivated in liquid Nutrient Broth (NB) medium at 180 rpm and 28 °C for 2–3 days until they reached the stationary phase. They were diluted at a factor of 1:100 in fresh NB medium and cultivated overnight. These starting cultures were used for experiments in their exponential growth phase. Bacteria of the genus *Streptomyces* were cultured in baffled flasks to avoid cell clumping.

Fungi were cultivated on cellophane membranes (Einmachfolie 1–2-3, Deti GmbH, Germany) on MMNC agar. Since all fungi used in the in vitro assays were non-sporulating, they were inoculated with mycelium pieces of defined sizes (~ 19.6 mm^2^). They were grown for 7–10 days at 25 °C in the dark. Mycelial pieces obtained from parts of the actively growing mycelium were used for inoculation.

### Sequencing of the 16S rRNA gene or ITS region for identification of the isolates

A total of 390 bacterial and 39 fungal isolates were selected based on distinct morphological characteristics and were identified at the Genus level to allow for broad phylogenetic classification. For identification of bacteria, colony PCRs were performed. Due to the rigidity of *Streptomyces* colonies, DNA was in this case extracted from liquid cultures using the DNeasy UltraClean Microbial Kit (Biotechnologies, Madison, USA), following manufacturer’s instructions, and 20–50 ng µL^−1^ were used for PCR. The v1-9 region of the 16S rRNA gene was amplified with the primers 27F (5′-AGAGTTTGATCCTGGCTCAG-3′) and 1492R (5′-ACGGYTACCTTGTTACGACTT-3′) [77, 78]. PCR amplification for bacteria was performed with an initial denaturation at 94 °C for 10 min, followed by 35 cycles of 94 °C for 30 s, 50 °C for 30 s, and 72 °C for 2 min, with final elongation at 72 °C for 5 min. For identification of fungi, DNA was extracted from 5 to 10 mg mycelium with the Animal and Fungi DNA Preparation Kit (Jena Bioscience, Germany) according to manufacturer’s instructions. The ITS region was amplified using around 100 ng µL^−1^ DNA and the primers ITS1-F (TCCGTAGGTGAACCTGCGG) and ITS4-R (TCCTCCGCTTATTGATATGC) [79]. For fungi, amplification was performed with an initial denaturation at 95 °C for 5 min, followed by 35–40 cycles of 95 °C for 30 s, 51.5 °C for 30 s and 72 °C for 45 s, and a final elongation at 72 °C for 7 min. PCR results were verified by agarose gel electrophoresis (1% agarose in 1× TAE buffer, 0.005% Midori Green, loading dye 3× OrangeG, 180 V, 37 min). The unpurified PCR products were sent for unidirectional sequencing (Eurofins genomics, Germany, seq2plate kit). The sequences were subjected to BLAST analysis using NCBI (https://blast.ncbi.nlm.nih.gov/Blast.cgi). Sequences with > 97% identity were assigned to the respective genera. The NCBI GenBank accession numbers for the bacterial isolates are PV918716-PV918751 (Table S2), and for the fungal isolates PV918686-PV918701 (Table S3). *Lycoperdon* sp. F31 was identified morphologically at the Genus level and was not sequenced.

### Identification of potentially beneficial isolates based on different in vitro assays

36 bacterial and 17 fungal isolates were screened for their plant growth-promoting and abiotic stress resistance abilities in different in vitro assays, regardless of their origin (tree species/site). Due to the high number of isolates, the selection of bacteria and fungi for characterization and screening within in vitro assays was based on the 16S and ITS rRNA gene/region sequencing results and literature research. Genera that were already known to contain plant growth-promoters, specifically in spruce, were preferably, albeit not exclusively, selected. Bacterial starting cultures were prepared as described above. Unless otherwise stated, cells were harvested by centrifugation (5,000 xg, 5–10 min) and washed twice in 1× PBS before each assay. Fungi were grown on MMNC agar plates as described above, with pieces of actively growing mycelium used for inoculation during the assays. All in vitro assays were performed in three biological and three technical replicates. We established a quantitative scoring system based on the performance of the isolates in standardized in vitro assays to identify and describe strains with outstanding performance in specific or multiple traits. Scores from 0 to 3 were assigned for each assay, with higher scores indicating a better performance. For bacteria, a maximum cumulative score of 24 was possible (across three stress tolerance and five plant growth promotion assays), and for fungi, the maximum was 12 (across two stress tolerance and two plant growth promotion assays). Scores were assigned as either 0 or 3 for binary assays (siderophore production, ACC deaminase production, nitrogen fixation, phosphate solubilization) or on a scale from 0 to 3 based on defined thresholds for semi-quantitative assays (IAA production and stress tolerance assays for bacteria and fungi, phosphate solubilization for fungi, Table S4).

#### Siderophore production

For the detection of siderophore production, the Chrome Azurol S (CAS)-overlay agar method of [80] and [81] was used with modifications. After cell washing, the optical density at 600 nm (OD_600_) was measured and adjusted to 0.5. 50 µL of each bacterial suspension was spotted on NB agar plates and incubated at 28 °C. After 48 h, 10 mL of the CAS-overlay agar (CAS (Sigma Aldrich, USA), FeCl_3_ (Fluka Biochemika, Germany), HDTMA (Sigma Aldrich, USA) mixed 1:10 with 32.24 g L^−1^ Piperazin-N’N-bis-(2-ethanesulfonic acid) in 0.9% agar, pH 6.8) was pipetted onto the bacterial colonies. Siderophore production was visible after 12 h by a color change from blue to orange.

#### 1-Aminocyclopropane-1-carboxylate deaminase production

The enzyme 1-aminocyclopropane-1-carboxylate (ACC) deaminase stimulates plant growth by hydrolyzing the ethylene precursor ACC [39]. Bacterial growth on minimal medium with ACC as single nitrogen source indicates the presence of an ACC deaminase. The assay was performed according to [82] and [83]. The OD_600_ of the bacterial suspensions was adjusted to 0.5. 25 µL were spotted on M9 medium with 9.35 mM NH_4_Cl, 3 mM ACC (Thermo Fisher Scientific, Waltham, MA, USA) or no nitrogen source. After incubation at 28 °C for 10 days, the utilization of ACC as nitrogen source was determined by comparing bacterial growth on the three different media. The strain *Variovorax* sp. M92526_27 [38] was used as an ACC-utilizing positive control.

#### Identification of potentially nitrogen-fixing bacteria

Two different media were used for the identification of nitrogen-fixing bacteria. Nitrogen-free semi-solid Nfb-agar was prepared according to [84]. Cell suspensions were adjusted to an OD_600_ of 0.1 and 10 µL were spotted onto 5 mL of Nfb-agar in glass tubes. After incubation for 144 h, fixation of atmospheric nitrogen was visible by the formation of pellicles within the agar. Single bacterial colonies were additionally streaked out on nitrogen-free Jensen’s agar (in g L^−1^: sucrose, 20; K_2_HPO_4_, 1; MgSO_4_ · 5 H_2_O, 0.5; NaCl, 0.5; FeSO_4_ · 7 H_2_O, 0.183; Na_2_MoO_4_, 0.005; CaCO_3_, 2) according to [38]. Colony growth indicates fixation of nitrogen. The nitrogen fixation assay was only considered positive if bacteria were able to grow on both media. *Azospirillum brasilense* Sp7 was used as a positive control.

#### Phosphate solubilization

Phosphate solubilization was tested on National Botanical Research Institute’s phosphate (NBRIP) growth medium (in g L^−1^: glucose, 10; Ca_3_(PO_4_)_2_, 5; MgCl_2_ · 6 H_2_O, 5; MgSO_4_ · 7 H_2_O, 0.25; KCl, 0.2; (NH_4_)_2_SO_4_, 0.1; pH 7.0) according to [85]. For bacteria, the OD_600_ was adjusted to 0.5 and 25 µL were spotted on NBRIP agar plates. After incubation at 28 °C for 10 days, phosphate solubilization was visible by formation of a clear zone around the bacterial spots. Phosphate-solubilizing *Luteibacter* sp. Cha2324a_16 [38] was used as a positive control. For fungi, sterile cellophane membranes were added on the NBRIP agar surface and inoculated with mycelial pieces. Plates were incubated for 10 days at 25 °C in the dark. For evaluation, the cellophane membranes were removed to reveal the clear halos. Some halos were clearer than others and some were faintly visible, which accounted for the different scores (0–3) assigned in the case of fungi.

#### Production of indole-3-acetic-acid

The production of indole-3-acetic acid (IAA) was determined by the colorimetric method of [86]. Bacterial pre-cultures were prepared as described above and transferred into NB liquid medium with and without 1.5 mg mL^−1^ of the IAA-precursor L-tryptophan (Carl Roth, Germany). They were incubated for 72 h at 28 °C and 180 rpm in the dark. The OD_600_ was adjusted to a uniform value (lowest OD_600_ measured). Cells were harvested at 5,000 xg for 2 min and 100 µL of the supernatant was mixed with 100 µL of Salkowski reagent (0.01 M FeCl_3_ anhydrous in 35% perchloric acid) and 1 µL orthophosphoric acid in a 96 well plate. After 40 min of incubation, the IAA content was measured at 530 nm in a plate reader (SpectraMax iD3, Molecular Devices). Commercial indole-3-acetic acid (Fluka Biochemika, Germany) was used at concentrations from 0 to 100 µg mL^−1^ to generate a standard curve. For data evaluation, the OD_600_ was normalized to 1. *Herbaspirillum frisingense* GSF30 served as a positive control. IAA amounts > 5 µg mL^−1^ were considered as positive IAA-production. For fungi, 3 mL MMNC liquid medium with and without 1.5 mg mL^−1^ L-tryptophan was inoculated with 2 mycelial pieces in 24-deep-well plates. After incubation for 96 h, 100 µL of the supernatant was mixed with 100 µL of Salkowski reagent and 1 µL orthophosphoric acid in 96-well plates and the assay was performed as described above. For evaluation, IAA-production was normalized to the fungal dry biomass. Values above a threshold value of 0.5 µg mg^−1^ were considered as positive IAA-production.

#### Tolerance to different abiotic stresses

To study their tolerance to different abiotic stresses (salt, osmotic, pH), 2 µL of the bacterial main cultures were transferred into 200 µL NB liquid medium with seven different concentrations of either NaCl, or PEG, and H^+^ (Table S4). NaCl was used for salt stress induction (modified from [87]), and different amounts of polyethylene glycol 6000 (PEG, Serva Electrophoresis GmbH, Germany) were applied to measure osmotic stress tolerance. The amount of PEG was based on decreasing water potentials using the formula of [88], according to [89] and [90], with 326 g L^−1^ PEG creating an osmotic pressure of − 1.2 MPa. With a pH value of around 5, forest soils are relatively acidic [91]. Therefore, bacterial tolerance to low pH values was also investigated. Tolerance to different pH values was analyzed by adjusting the pH of NB liquid medium using NaOH and HCl (modified from [38]). All assays were performed in sterile 96-well plates and the OD_600_ was measured after 0, 24 and 48 h. Bacterial recovery from the 2–3 highest concentrations of NaCl, PEG and H^+^ was determined by plating 25 µL of these concentrations on NB agar plates, incubating them at 28 °C for 48 h, and observing bacterial growth. Fungi were subjected to salt and osmotic stress. Tolerance to low pH values was not tested, since most fungi grow at a pH range of 5 to 6 [92], matching the demands for growth on forest soils. NaCl tolerance was tested on agar plates containing MMNC medium with seven different NaCl concentrations (Table S4). They were grown for 10 days at 25 °C in the dark. The agar surface area covered by fungal mycelium was measured for each plate using ImageJ. PEG tolerance was assessed in liquid, adding 2 mycelial pieces to 3 mL MMNC medium with seven different PEG concentrations (Table S4). They were incubated for 96 h at 130 rpm and 25 °C. The mycelium was harvested and washed thoroughly 2 × in ddH_2_O. Mycelia were dried at 65 °C and the dry weight was measured.

### Optimized 24-well plate assay for in planta screening of 29 bacterial isolates for growth promotion of *Picea abies* under drought and well-watered conditions

**Fig. 1:**
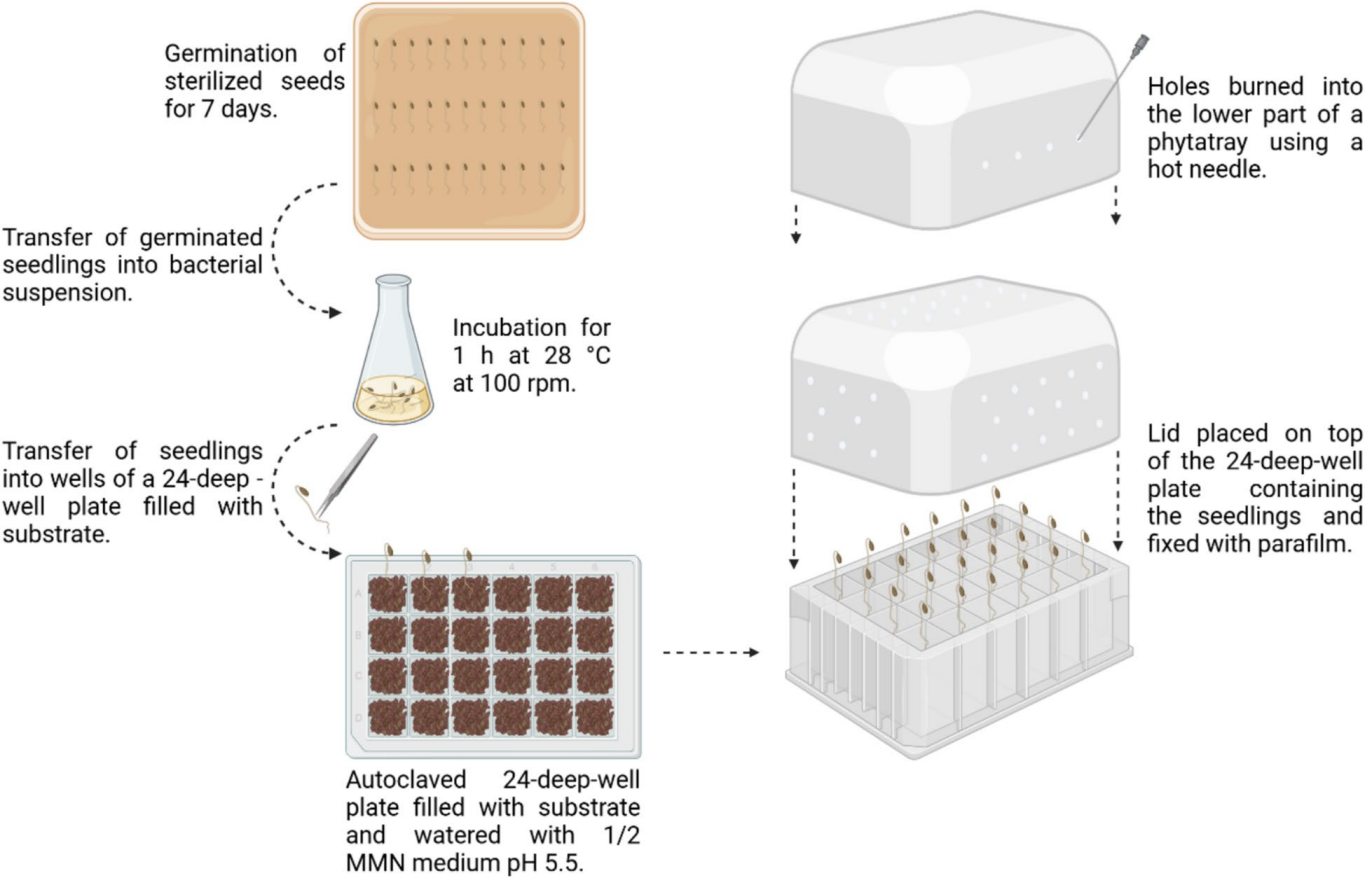
Optimized screening system for PGPMs in spruce using 24-deep-well plates. *P. abies* seeds are germinating for 1 week on MMNC agar plates before they are inoculated in bacterial suspensions or 1× PBS (control) for 1 h. They are transferred into 24-deep-well plates filled with sterilized soil (n = 24 for each treatment). After burning holes into the lower part of a phytatray with a hot needle, plates are sealed with the modified lid and put into a phytochamber for 3 weeks. This figure was created with BioRender.com

An axenic 24-well test system was developed to examine the effect of bacteria characterized in the in vitro assays on the growth of *P. abies* seedlings (Fig. 1). *P. abies* was chosen because its seeds can be readily sterilized and it rapidly develops into small, experimentally manageable seedlings. To set up the system, 24-deep-well plates were filled with custom-made substrate (“TS1 fein” with white peat (0–5 mm), perlite (1–7.5 mm) and black peat, 3:1:1 (v/v), pH 5.5, without NPK-fertilizer; Klasmann-Deilmann GmbH, Germany). For well-watered conditions, 3.25 mL ddH_2_O and for drought conditions, 0.75 mL ddH_2_O were added to all wells. After autoclaving, 0.5 mL ½ MMN (MMNC without carbon source) pH 5.5 liquid medium was added to each well. *P. abies* seeds were obtained from the Bavarian State Forests Nursery (Bayerische Staatsforsten AöR, Laufen, Germany). They were washed in ddH_2_O for 30 min and sterilized in 35% H_2_O_2_ for 45 min, rinsed with ddH_2_O for 30 min, and placed on MMNC square petri dishes for germination for 1 week in the dark in a tilted position. To inoculate the seedlings, bacterial main cultures were washed twice in 1 × PBS, and the cell density was adjusted to 10^8^ cells per mL. Seedlings were incubated in the bacterial suspension for 1 h at 28 °C and 100 rpm. Each inoculated seedling was transferred into one well of the prepared 24-well plates. The lower part of a phytatray (Sigma-Aldrich, Munich, Germany) was used as a translucent cover for the 24-well plates. To enable gas exchange, small holes were burned into the lid using a hot needle. The lid was secured to the plates with parafilm. The 24-well test systems were kept in a phytochamber under controlled conditions (8/16 day/night cycle, 55% humidity, 20 °C, 25 Wm^−2^ light intensity). After 3 weeks, plant morphology was evaluated by measuring root and shoot fresh weight and length, as well as entire seedling fresh weight and length, and seedling dry weight after drying the seedlings for 3 weeks at room temperature. Moreover, bacterial rhizosphere competence was estimated by counting colony forming units (CFUs) after 3 weeks of plant growth in a phytochamber. To this end, three roots per treatment (inoculated/non-inoculated, drought-stressed/well-watered) were grinded in a sterilized mortar in 1 × PBS and diluted up to a factor of 10^–4^. 100 µL of the 10^–3^ and 10^–4^ dilution were plated on NB medium, and the CFUs were counted after 24–48 h incubation at 28 °C. Only plants with vital root tips were selected for evaluation.

In the initial experiments using the 24-well test system, internal controls were included on each plate to monitor potential variances between the plates. Six control plants and twelve inoculated plants were used, with one row of six wells left empty between the two treatments to prevent potential cross-contamination. No variation between the control plants was observed during the plant experiments. Consequently, to increase the number of replicates, the internal controls were omitted, and the control plants were placed in a separate plate, allowing for 24 replicates for both the control and inoculated plants. It was demonstrated that the internal controls remained consistent across different plates (Figure S1).

### Statistical analysis

All figures (except those relating to microbiome data) were generated using Origin Pro (version 2021b). Plant parameter data were tested for normality using the Shapiro–Wilk test. If normal distribution was not confirmed, a non-parametric Mann–Whitney test was used. Statistical differences between inoculated treatments and their respective control groups were assessed using one-way ANOVA followed by Fisher’s Least Significant Difference (LSD) test at a significance level of p = 0.05. Details of the statistical analysis of amplicon sequencing data are provided in the relevant section.

## Results

### The resident root-associated microbial community at both sites and tree species bears the potential for isolating PGP and stress resistant microbes

**Fig. 2:**
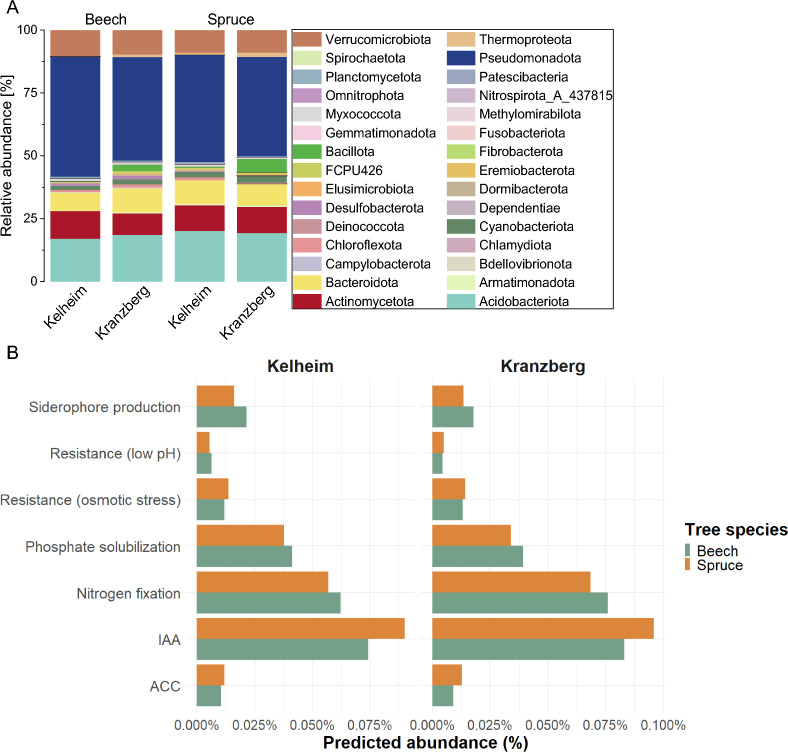
Composition of bacterial phyla and predicted functional traits of rhizosphere communities associated with beech and spruce in Kelheim and Kranzberg. **A** Relative abundance [%] of the bacterial phyla in beech and spruce rhizosphere communities in Kelheim and Kranzberg. Community composition was evaluated using the microbiome packages (v1.24.0, [69]) in R. Bray–Curtis distance was calculated using the R package vegan (v2.6.6, [70]). The plot was generated using Origin Pro (version 2021b). **B** Predicted abundance [%] of different enzymes for the bacterial beech and spruce rhizosphere community in Kelheim and Kranzberg being involved in the PGP and stress resistance pathways evaluated in the in vitro assays of this study. Tax4Fun2 [71] was used to predict the functional profiles based on the default database and a 97% similarity cut-off. Predicted enzymes were manually inspected to identify those involved in the pathways evaluated in the in vitro assays. Data visualization was performed using the ggplot2 package [72]

Given that both chosen sampling sites (Kranzberg and Kelheim) are (moderately) dry, environmentally distinct and host two different tree species, the resident root-associated microbial community at the time of sampling after one week of low or no precipitation (Table S1) was analyzed by amplicon sequencing to visualize the diversity present and to evaluate its potential for isolating PGP and stress resistant candidates. A total of 3,364 Amplicon sequence variants (ASVs) for bacteria, and 4,357 ASVs for fungi were found at both sites. The resident root-associated microbial community was highly similar across both sites and tree species, with only minor differences. The fungal community showed a separation by tree species as well as by site (Figure S3 B), while differences in the bacterial community composition were influenced more by tree species than by sampling site (Figure S3 A). The main difference between sites was a higher relative abundance of Bacillota in Kranzberg ASVs, which was even more pronounced for spruce compared to beech (Fig. 2 A). In the case of fungi, Mortierellomycota were highly represented in spruce in Kranzberg. In Kelheim, a higher abundance of Basidiomycota was detected compared to Kranzberg (Figure S2).

A total of 30 bacterial phyla were identified in the rhizosphere microbiome (Fig. 2 A). The top 10 most abundant phyla comprised Pseudomonadota, Acidobacteriota, Actinomycetota, Verrucomicrobiota, Bacteroidota, Bacillota, Cyanobacteriota, Chloroflexota, Thermoproteota and Desulfobacterota (in descending order). Overall, 15 different phyla were found in the fungal rhizosphere microbiome (Figure S2). Ascomycota was the dominant phylum, followed by Basidiomycota. Mortierellomycota was the third most represented phylum.

The bacterial amplicon sequencing data was further subjected to a functional prediction analysis to provide an initial characterization of the microbial community and to assess its potential for identifying PGP and stress resistant isolates (Fig. 2 B). The trait with the highest predicted abundance was IAA-production followed by N-fixation. The potential to harbor bacteria with the desired traits (PGP and stress resistance) was similar across both sites and tree species.

### Isolation and identification of root-associated microorganisms

A total of 1,292 bacteria and 59 fungal isolates were obtained from beech and spruce rhizosphere soil in Kranzberg (moderate-dry site) and Kelheim (dry site). Bacteria were classified into about 20 morphotypes based on their morphology on agar plates. From each morphotype, between 10 and 20 strains were subjected to Sanger sequencing, resulting in the identification of 390 bacteria belonging to 25 different genera. Overall, 230 bacteria originated from Kranzberg (23 different genera) and 160 from Kelheim (15 different genera) (Table 1). The bacterial isolates were affiliated with four different phyla: Pseudomonadota (n = 194), Bacillota (n = 143), Actinomycetota (n = 31) and Bacteroidota (n = 4). Genera represented by fewer than three isolates were classified as low abundant strains (n = 18). A small fraction of isolates (0.5%) was assigned to paraphyletic groups. The most frequently isolated genus was *Paraburkholderia* with 121 isolates, followed by *Bacillus* with 43 isolates, and *Caballeronia* and *Paenibacillus* with 34 isolates. *Bacillus* was isolated more often in Kranzberg than in Kelheim (38 vs 5). In Kelheim, *Paraburkholderia* accounted for 45% (n = 72) of all genera, while in Kranzberg, a more even distribution of the different bacterial genera was observed. In addition to bacteria, 39 fungi were identified to the Genus level, with 25 isolated from Kelheim and 14 from Kranzberg (Table S5). Their Genus-level identification revealed 14 different genera (13 in Kelheim, 4 in Kranzberg) belonging to 3 different phyla (22 Ascomycota, 15 Mucoromycota, 2 Basidiomycota). *Penicillium* and *Umbelopsis* were the most frequently isolated fungal genera with 8 isolates each, followed by *Mortierella* with 4 isolates.

**Table 1:** Bacterial phyla and genera isolated as single strains from beech and spruce roots in Kelheim and Kranzberg

Phylum	Genus	Number of isolates				
		Kranzberg		Kelheim		Sum
		Beech	Spruce	Beech	Spruce	
Pseudomonadota	*Paraburkholderia*	26	23	47	25	121
	*Caballeronia*	11	9	3	11	34
	*Pseudomonas*	20	3	3	3	29
	*Herbaspirillum*	3	2	0	0	5
	*Collimonas*	0	0	0	5	5
Bacillota	*Bacillus*	18	20	3	2	43
	*Viridibacillus*	12	4	1	4	21
	*Peribacillus*	7	6	8	9	30
	*Psychrobacillus*	7	1	2	2	12
	*Paenibacillus*	17	6	1	10	34
	*Sporosarcina*	0	0	1	2	3
Actinomycetota	*Streptomyces*/ *Kitasatospora*	11	6	7	3	27
	*Rhodococcus*	0	0	3	1	4
Bacteroidota	*Chryseobacterium*	0	4	0	0	4
Low abundant strains		12	2	1	3	18
Sum		144	86	80	80	390

The designation “Low abundant strains” refers to genera with less than 3 isolates

### Screening of microbial isolates for their tolerance to abiotic stresses and plant growth-promoting activities

Since no substantial differences in the microbial rhizosphere communities were observed between sites or species (neither in terms of relative abundance nor functional prediction), the isolates were screened for their plant growth-promoting and abiotic stress resistance abilities in different in vitro assays, regardless of their origin (beech/spruce, Kelheim/Kranzberg). In total, 36 bacterial strains were characterized, of which 20 were isolated from Kelheim and 16 from Kranzberg (Table S2). Additionally, 17 fungal strains were selected for characterization, with 13 from Kelheim and 4 from Kranzberg (Table S3). The choice of isolates for further characterization was guided by literature research, trying to incorporate a diverse set of genera.

### Bacteria displayed a high PGP potential in vitro

The abiotic stress tolerance assays included different concentrations of NaCl, PEG, or different pH values and each tested strain was evaluated with a scoring system based on the test results (methods). *Streptomyces* sp. Ke434, *Rhodococcus* sp. Ke466 and *Streptomyces* sp. KF215 showed the highest tolerance, reaching a score of 7 out of 9 in the stress tolerance assays (Fig. 4A). *Streptomyces* sp. Ke434 exhibited tolerance to an osmotic potential of − 1.5 MPa, whereas *Rhodococcus* sp. Ke466 and *Streptomyces* sp. KF215 tolerated up to − 1.25 MPa (Table 2). The average PEG tolerance for bacteria was − 0.78 ± 0.24 MPa. The highest NaCl concentration was tolerated by two *Rhodococcus* strains (Ke442, Ke466), two *Streptomyces* strains (Ke434, KF143) and *Sporosarcina* sp. Ke477, withstanding concentrations of up to 5.5% (Table 2), while the average NaCl tolerance was 2.14 ± 1.96%. Almost all strains were able to grow at a pH of 5. Eight strains even grew at pH 4, including six *Paraburkholderia* strains (Ke15, Ke24, Ke162, Ke296, Ke341, Ke398), *Rhizobium* sp. Ke26 and *Caballeronia* sp. Ke57 (Table 2). The average pH tolerance was 4.79 ± 0.74. None of the strains was able to grow below a pH of 4. Most of the bacteria were also able to recover from the 3 highest stress levels (Table S6). Recovery from low pH values seemed to be most difficult, since only a few isolates could recover from pH values below 4, namely *Bacillus* sp. Ke157, *Rhodococcus* sp. Ke466 and *Viridibacillus* sp. KF108, which recovered after incubation in NB medium with a pH of 2.

**Table 2:**
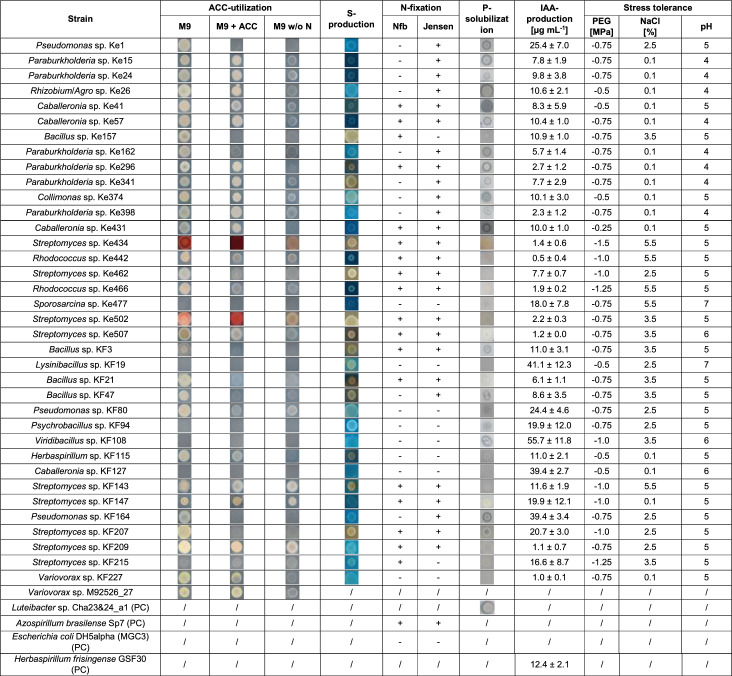
Summary of all bacterial in vitro assays

For ACC-utilization, siderophore production (S-production), and P-solubilization assays, representative colony pictures are shown. For the N-fixation assay, “+” indicates detectable pellicle formation or colony growth on agar, while “−” indicates absence of pellicles/growth. For IAA-production, the mean amount of IAA ± standard deviation (n = 3) is shown. For the stress tolerance assays, the lowest osmotic pressure, the highest NaCl concentration and the lowest tolerated pH allowing visible growth are given. All assays were performed using 3 biological and 3 technical replicates. The positive controls (PC) *Variovorax* sp. M92526_27 (ACC utilization), *Luteibacter* sp. Cha2324a_16 (phosphate solubilization), *Azospirillum brasilense* Sp7 (nitrogen fixation) and *Herbaspirillum frisingense* GSF30 (IAA-production) were only tested in their respective assays. “/”  = not tested

In addition to stress tolerance, the plant growth-promoting (PGP) potential was also evaluated by assessing the bacteria’s ability to solubilize phosphate, produce siderophores, fix nitrogen, utilize ACC, and produce IAA. *Caballeronia* sp. Ke41, *Paraburkholderia* sp. Ke296 and *Streptomyces* sp. KF207 showed the highest PGP abilities with scores of 13 and 12 out of 15 (Table 2; Fig. 4A). 16 of 36 strains (~ 44%) were able to produce siderophores, particularly from the genera *Bacillus* and *Streptomyces*. Solubilization of phosphate was detected in 19 of 36 strains (~ 53%) and included all *Paraburkholderia* strains. ACC-utilization was observed in 20 strains (~ 56%), including all *Paraburkholderia* and most *Streptomyces* strains. Regarding nitrogen fixation, 16 of 36 strains (~ 44%), especially represented by *Rhodococcus* and *Streptomyces*, showed growth in both tested nitrogen-free media, indicating their nitrogen-fixing potential. All bacteria were also examined for their ability to produce the phytohormone indole-3-acetic acid (IAA). 28 out of the 36 strains (78%) produced IAA at concentrations above the threshold level of 5 µg mL^−1^ (Table 2). 11 strains produced IAA concentrations above 15 µg mL^−1^ (score of 3) with *Viridibacillus* sp. KF108, *Lysinibacillus* sp. KF 19, *Caballeronia* sp. KF127, and *Pseudomonas* sp. KF164 reaching the highest concentrations of 55.7, 41.1, and 39.4 µg mL^−1^ (Table 2). The positive control *Herbaspirillum frisingense* GSF30 produced 12.4 ± 2.1 µg mL^−1^ IAA. Some of the tested strains even showed an IAA-production above the threshold level of 5 µg mL^−1^ after growth without tryptophan (Table S7).

**Fig. 3:**
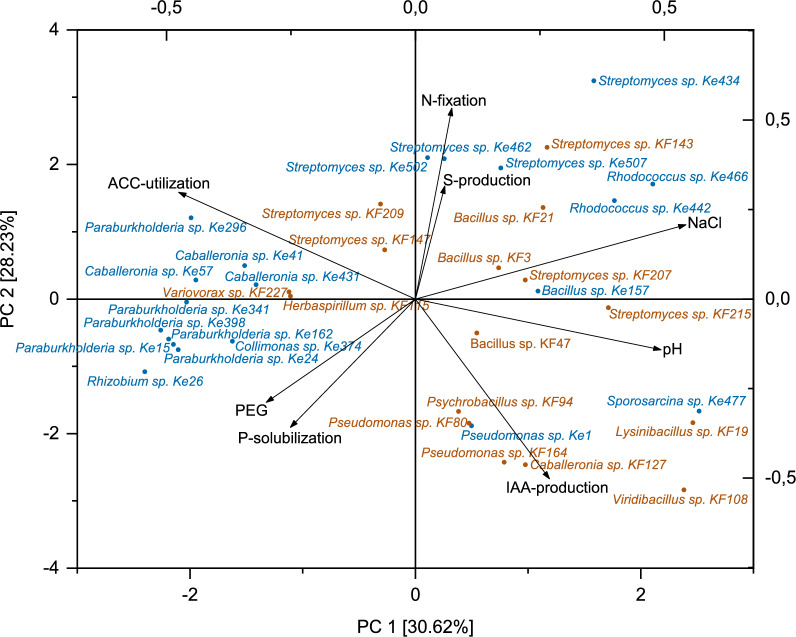
Principal Component Analysis (PCA) identifying genus-related clusters for the in vitro assays of the bacterial strain collection. Variables: pH, PEG and NaCl tolerance, as well as production of IAA based on measured values; production of siderophores (S-production), P-solubilization, ACC-utilization and N-fixation based on the presence/absence of the PGP trait (1/0). Blue: Kelheim-derived isolates. Brown: Kranzberg-derived isolates

**Fig. 4:**
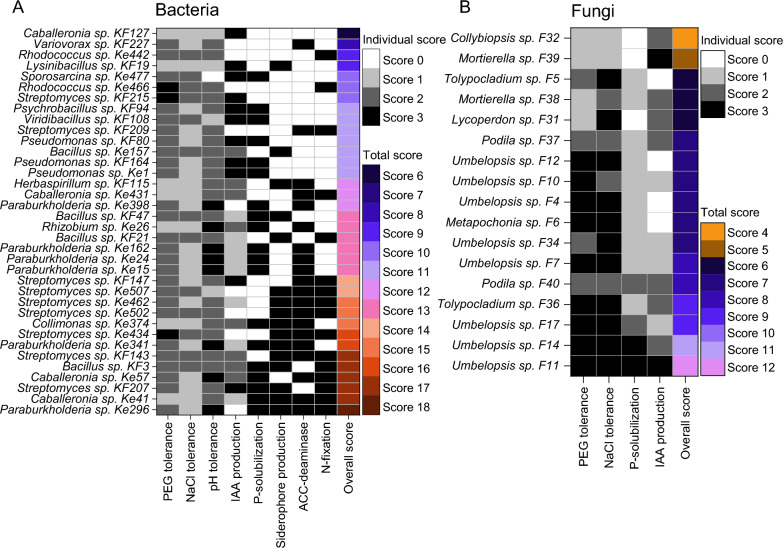
Heatmaps showing the scoring of the isolates in the different in vitro assays. The isolates are sorted by ascending overall scores, given in the column on the right. **A** For bacteria PEG-, NaCl- and pH-tolerance, as well as IAA-production were evaluated with scores ranging from 0 to 3. P-solubilization, siderophore-production, ACC-utilization and N-fixation were either evaluated with 0 for “not detectable” or 3 for “detectable”. All underlying bacterial in vitro assays were performed in 3 biological and 3 technical replicates. **B** For fungi PEG- and NaCl-tolerance, as well as P-solubilization and IAA-production were evaluated with scores ranging from 0 to 3. The underlying fungal stress tolerance assays were performed in 3 replicates, and fungal P-solubilization and IAA-production were performed in 4 replicates

Overall, 31 out of 36 strains (86%) exhibited PGP abilities in at least two different in vitro assays, while only five strains showed positive results in just one PGP assay (Table 2; Fig. 4 A). The combined results of the bacterial in vitro assays were subjected to a Principal Component Analysis (PCA) to identify potential genus-specific commonalities, which appeared as clusters (Fig. 3). A cluster consisting mainly of the genera *Paraburkholderia* and *Caballeronia* was represented by strong osmotic stress tolerance (PEG), as well as P-solubilization and ACC-utilization abilities. Actinomycete isolates of the genera *Streptomyces* and *Rhodococcus* clustered by comparably strong N-fixation, siderophore production and salt stress tolerance abilities. Since the isolated genera were not equally distributed between the two different sites, it is not possible to conclude on site-related effects.

*Streptomyces*, *Paraburkholderia*, *Caballeronia*, *Collimonas*, *Rhodococcus* and *Bacillus* ranked among the ten best performing bacterial genera of the in vitro assays. With the exception of *Collimonas* and *Rhodococcus*, the best performers belonged to the most abundant genera of the strain collection, highlighting a high prevalence of PGPB among the isolates.

### Fungi tolerated especially high NaCl and PEG concentrations

Fungal stress tolerance at different NaCl and PEG concentrations was generally high, with 8 out of 17 strains (47%) reaching the maximum score of 6 (Fig. 4B). The average osmotic tolerance was at − 1.13 ± 0.49 MPa. *Metapochonia* sp. F6 and three *Umbelopsis* strains (F11, F12, F14) grew well even at the highest PEG concentration tested (− 1.75 MPa) (Table 3). *Metapochonia* sp. F6 showed a high dry weight (DW) at all PEG concentrations, with a maximum DW of 37.4 ± 6.6 mg at − 1.0 MPa. Since several other *Umbelopsis* strains, such as F10, F11, F12, F14 and F17, had similarly elevated DWs, this might indicate a potential usage of PEG as a carbon source by these strains. The average NaCl tolerance was at 6.07 ± 3.03%. *Metapochonia* sp. F6 exhibited the highest detectable salt tolerance, growing at 12% NaCl, followed by ten strains that grew at up to 7.5% NaCl (Table 3).

**Table 3:**
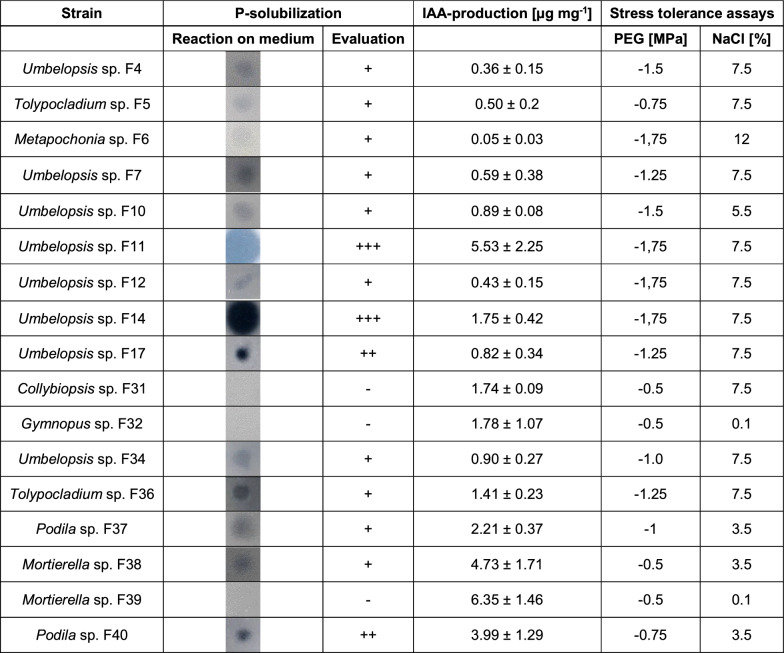
Summary of all fungal in vitro assays

For P-solubilization assays, representative colony pictures are shown (n = 4). Some halos observed in the P-solubilization assay were clearer and others only lightly indicated, being responsible for the different scores assigned (0–3). For IAA-production, the mean amount of IAA ± standard deviation (n = 4) is shown. For the stress tolerance assays, the lowest osmotic pressure and the highest NaCl-concentration allowing visible growth are given. Stress tolerance assays were performed in 3 replicates

Regarding their PGP potential, almost all fungi solubilized phosphate (14 of 17, ~ 82%), although to different extents (Table 3), and 13 out of 17 fungi (~ 77%) produced IAA above the threshold value of 0.5 µg mg^−1^ (Table 3). Two fungi showed an especially high IAA-production: *Umbelopsis* sp. F11 with 5.5 ± 2.3 µg mg^−1^ and *Mortierella* sp. F39 with 6.4 ± 1.5 µg mg^−1^, both reaching the maximum score of 3 for IAA-production. In contrast to bacteria, fungi were not able to produce IAA without tryptophan (Table S8).

Among the bacterial isolates, *Paraburkholderia* sp. Ke296 reached the highest overall score (18), followed by two *Caballeronia* strains (Ke41 and Ke57), two *Streptomyces* strains (KF147 and KF207) and *Bacillus* sp. KF3, each with a score of 17 (Fig. 4A). Two fungal isolates, *Umbelopsis* sp. F11 and *Umbelopsis* sp. F14 reached the highest scores of 12 and 11, respectively (Fig. 4B).

### Application of bacteria to a newly developed 24-well plate test system reveals two bacteria promoting spruce growth under drought

Since in vitro assays do not directly reflect real-life conditions, they can serve well as a preliminary screening tool, but cannot reliably predict functional effects under natural or field conditions. To address this, *in planta* experiments are of high importance and thus a rapid and axenic screening system was established using the plant of interest (spruce). Specifically, a 24-well plate assay was developed to efficiently test the pre-selected isolates for their potential to promote seedling drought tolerance (Fig. 1 and methods).

Pre-experiments showed neglectable variation between plates, and controls for cross-contamination were consistently negative. The final plate design allowed each selected isolate to be tested on 24 individual seedlings under control and drought conditions, respectively. Re-isolation 3 weeks after inoculation revealed colonization rates ranging from 4.9 × 10^3^ to 7.7 × 10^7^ colonies per mg root except for *Psychrobacillus* sp. KF94 and *Viridibacillus* sp. KF108, which could not be re-isolated (Table S9).

**Fig. 5:**
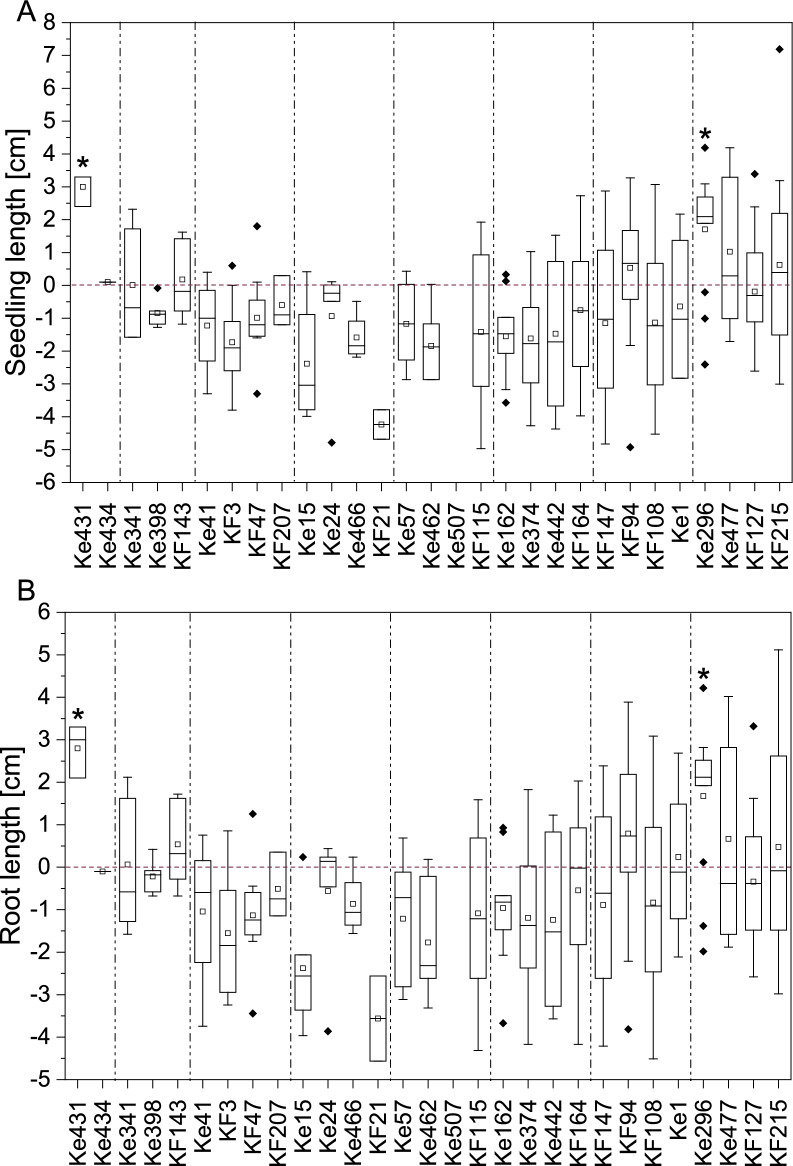
Seedling growth performance under drought. Displayed are the absolute changes in **A** entire seedling length and **B** root length under drought stress after inoculation with individual bacterial isolates. The red zero baseline represents the mean of the uninoculated control within each experiment. Positive values indicate an absolute increase, and negative values a decrease in length compared to the respective control. Individual experiments are separated by dashed lines. Significant plant growth-promotion after inoculation with the isolates compared to the respective control group is marked with asterisks. Statistical differences between groups were assessed using one-way ANOVA followed by Fisher’s Least Significant Difference (LSD) test at a significance level of *p* = 0.05. In the first three experiments (inoculation with Ke431, Ke434, Ke341, Ke398, KF143, Ke41, KF3, KF47, KF207), 12 replicates were used for inoculated plants. For the remaining 5 experiments, 24 replicates were used. Control plants were always tested with 24 replicates

29 of the 36 bacterial isolates were tested in the 24-well system. Several showed significant positive effects on seedling growth under well-watered (WW) conditions (Table S10). However, two isolates clearly stood out due to their exceptionally pronounced PGP effects under drought stress (DS): *Caballeronia* sp. Ke431 and *Paraburkholderia* sp. Ke296. Both significantly increased seedling length under DS conditions, mainly by an increase in root length, compared to the uninoculated controls (Fig. 5A, B). Both strains were even able to increase root length compared to the WW control (Figure S4). Strikingly, *Caballeronia* sp. Ke431 increased seedling survival threefold and *Paraburkholderia* sp. Ke296 maintained survival (1.2-fold increase), both under DS (Table S9). Moreover, although *Psychrobacillus* sp. KF94 and *Viridibacillus* sp. KF108 could not be re-isolated from spruce roots, seedling survival increased about threefold after inoculation with the two isolates, and *Psychrobacillus* sp. KF94 also significantly increased seedling dry weight under DS (Figure S5 B), but had no other beneficial effects on plant growth (Table S10).

**Fig. 6:**
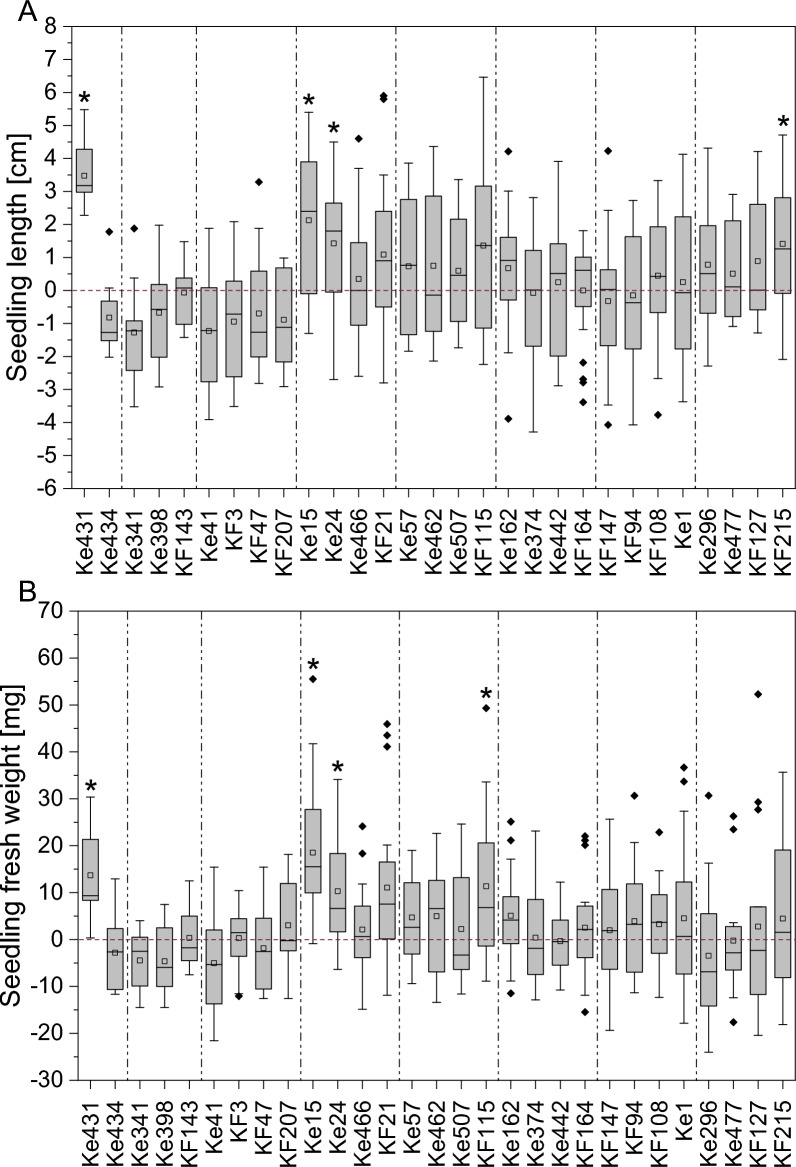
Seedling performance under well-watered conditions. Displayed are the absolute changes in **A** entire seedling length and **B** seedling fresh weight under well-watered conditions after inoculation with individual bacterial isolates. The red zero baseline represents the mean of the uninoculated control within each experiment. Positive values indicate an absolute increase, and negative values a decrease in length or fresh weight compared to the respective control. Individual experiments are separated by dashed lines. Significant plant growth-promotion after inoculation with the isolates compared to the respective control group is marked with asterisks. Statistical differences between groups were assessed using one-way ANOVA followed by Fisher’s Least Significant Difference (LSD) test at a significance level of *p* = 0.05. In the first three experiments (inoculation with Ke431, Ke434, Ke341, Ke398, KF143, Ke41, KF3, KF47, KF207), 12 replicates were used for inoculated plants. For the remaining 5 experiments, 24 replicates were used. Control plants were always tested with 24 replicates

*Caballeronia* sp. Ke431 additionally significantly improved seedling and root length as well as seedling, root and shoot fresh weight under WW conditions (Fig. 6, Figure S6, Figure S7 A, Figure S8 A).

Under WW conditions, also several other strains showed significant seedling growth-promoting effects (Table S10). Inoculation with *Paraburkholderia* sp. Ke15 and Ke24 significantly increased seedling and root length, seedling, root and shoot fresh weight and seedling dry weight (Fig. 6, Figure S5 A, Figure S6, Figure S7 A, Figure S8 A). Inoculation with *Streptomyces* sp. KF215 significantly enhanced seedling length, root length and root fresh weight (Fig. 6 A, Figure S6 and Figure S7 A). *Paraburkholderia* sp. Ke162 inoculation increased root fresh weight (Figure S7 A) and *Herbaspirillum* sp. KF115 showed significantly increased seedling, root and shoot fresh weight as well as dry weight (Fig. 6B, Figure S5 A, Figure S7 A, Figure S8 A). Inoculation with *Rhodococcus* sp. Ke466 and *Bacillus* sp. KF21 significantly increased seedling dry weight (Figure S5 A).

## Discussion

### The resident root-associated community bears a substantial potential for stress-resistant and plant growth-promoting microbes

The main objective of the present study was to isolate and identify bacteria and fungi with the potential to promote tree seedling growth under drought stress. Initially, a phylogenetic and functional characterization of the microbial rhizosphere community was performed to explore the population diversity available for isolation and to estimate its trait potential. To capture a broad spectrum and increase the probability of isolating drought-adapted microorganisms, sampling was conducted at two different (moderately) dry sites in the rhizosphere of spruce and beech after a period with low or absent precipitation (Table S1), as microorganisms can rapidly adapt to changes in environmental conditions [93]. Amplicon sequencing combined with the functional prediction analysis revealed a rhizosphere microbiome rich in taxa with known plant growth-promoting (PGP) capabilities, with only minor compositional differences observed between the two sites and tree species. For example, the bacterial phyla Actinomycetota and Pseudomonadota include many PGP and stress-resistant genera, such as *Streptomyces, Rhodococcus, Caballeronia* or *Paraburkholderia*. Their established role as stress-resistant PGPB in spruce and other plants [39, 49, 54, 94, 95] positions these genera as promising candidates for the objectives of this study, and representatives of all of them were also successfully isolated.

As anticipated, the amplicon sequencing data represented a much higher diversity compared to the strain collection, as isolation success is strongly influenced by culture conditions and the growth characteristics of individual taxa [96, 97]. Nevertheless, all four bacterial phyla identified in the collection (Bacteroidota, Actinomycetota, Bacillota, and Pseudomonadota) were also among the top 10 most abundant phyla in the microbiome data (Table 1, Fig. 2A). The genera *Paraburkholderia* and *Caballeronia* (both Pseudomonadota), which were isolated in high frequency, were also among the 8 most abundant genera in the bacterial rhizosphere community (Figure S11 A).

The bacterial diversity at both mixed spruce-beech study sites based on 16S rRNA gene amplicons is consistent with previously observed phyla distributions found at other spruce and beech sites, being dominated by Acidobacteria (now Acidobacteriota), Proteobacteria (now Pseudomonadota), Actinobacteria (now Actinomycetota), Verrucomicrobia (now Verrucomicrobiota), Bacteroidota, Firmicutes (now Bacillota), and Chloroflexi (now Chloroflexota) [98–103].

The observed fungal diversity was also equivalent to other studies on spruce and beech rhizosphere microbiomes, being dominated by Basidiomycota, followed by Ascomycota and Mucoromycota [99, 101, 103]. At the Genus level, the fungal spruce and beech rhizosphere microbiome is generally dominated by ectomycorrhizal genera such as *Cenococcum*, *Russula*, *Piloderma*, *Cortinarius*, *Hygrophorus* and (*Pseudo*)*Tomentella,* and also includes genera like *Archaeorhizomyces*, *Helotiales, Meliniomyces*, and *Penicillium* [22, 44, 101], all of which were among the 20 most abundant genera of the fungal microbiome observed in this study (Figure S11 B). The main fungal isolates in the present study were saprotrophic genera such as *Mortierella* and *Umbelopsis*, which were also reported in forest soils [102, 103]. Since the soil-dilution isolation method used in this study targets mainly root-associated saprotrophic fungi, isolation of genera belonging to the ectomycorrhiza was not to be expected, and complex isolation methods would be required to increase the number of these isolates [104].

### Microorganisms with high stress tolerance and plant growth-promoting abilities were identified in a classical in vitro screening

While isolates and amplicon data were initially obtained separately for each site and tree species, all samples were combined for the identification of PGP microbes. Given the minor differences in the microbial community observed between sites and tree species, the focus was placed on the total microbial community, enabling a comprehensive screening in vitro.

The strain collection of the present study exhibited multiple traits related to plant growth promotion, with 86% showing PGP abilities in more than one in vitro assay. *Paraburkholderia* sp. Ke296 reached the highest overall score and was tested positive in all PGP assays except for IAA-production, highlighting the high potential of this strain regarding plant growth promotion. When looking at the entire collection, however, IAA-production was the most prevalent PGP trait, as already predicted by the functional analysis. The IAA levels produced in pure culture can serve as an indicator of a strain's potential to mitigate the effects of osmotic stress on plant growth [105]. Almost half of all bacteria produced IAA above the threshold value of 5 µg mL^−1^ even in the absence of tryptophan (47%). Although the tryptophan-dependent pathway is the most common in bacteria, especially Gram-positive bacteria use alternative biosynthetic routes [106], which could thus partly be confirmed in the present study. Among the fungal isolates, 76% were capable of producing IAA. The highest IAA levels were observed in isolates belonging to the genera *Umbelopsis* and *Mortierella*, both of which are already known for their phytohormone production, including IAA [107–109].

Importantly, apart from expressing plant growth-promoting traits, strains must sustain these activities and exhibit stress tolerance under adverse conditions for effective use as PGPMs [110, 111]. The fungal isolates showed a particularly high stress tolerance with 47% of the strains growing on medium with high PEG (− 1.25, − 1.5, − 1.75 MPa) and NaCl concentrations (7.5, 12, 15%) represented by several *Umbelopsis* strains, *Tolypocladium* and *Metapochonia* (Table 3). So far, little is known about the salt tolerance of the fungi studied here. *Umbelopsis* strains isolated from pine were able to tolerate 3% NaCl, although their growth rate was reduced [112] and a *Tolypocladium* strain showed optimal spore production at up to 2% NaCl [113]. In comparison, the fungi tested in the present study showed a higher average NaCl tolerance of 6%. Moreover, some fungi of the present study showed even better growth on medium containing high compared to low PEG concentrations, suggesting they were able to biodegrade PEG and use it as a carbon source, as known for several microorganisms [114].

Members of the phyla Actinomycetota (*Streptomyces*, *Rhodococcus*) and Bacillota (*Viridibacillus*, *Bacillus* and *Sporosarcina*) showed the highest PEG and NaCl tolerance of the bacterial isolates. The high stress resistance observed for members of these phyla might be related to their ability to produce stress-resistant spores [115, 116]. *Streptomyces* sp. Ke434 exhibited the highest PEG and NaCl tolerance (− 1.5 MPa and 5.5%), making this strain an especially interesting candidate for a potential seedling protection against osmotic stress. Several studies have demonstrated that *Streptomyces* and *Rhodococcus* strains can grow at NaCl concentrations ranging from 2.6 to 5.5%, with a few rare examples capable of growing at concentrations as high as 10% NaCl [87, 117, 118]. The tolerated NaCl concentrations are remarkably high for bacteria, as concentrations of 3.5 to 5% NaCl are typically associated with slightly to moderately halophilic bacteria [119]. The highest PEG concentrations tested in this study approached the threshold for severe osmotic stress, as plants begin to wilt irreversibly at a soil water potential of − 1.5 MPa [120]. However, members of Bacillota and Actinomycetota are known to produce spores and osmoprotectants, enabling them to tolerate high osmotic stress [21, 111, 119].

Several PGP and stress resistance traits act synergistically and unfold their greatest potential in combination. Drought stressed plants inoculated with PGPB showed improved root and shoot growth and reduced cell damage that was connected with high IAA and ACC deaminase concentrations [21]. After inoculation with *Streptomyces* strains, beneficial effects on plant growth under NaCl and water stress were observed, such as production of high IAA and ACC deaminase levels, both of which correlate with abiotic stress alleviation in plants [116].

### Double screening in form of a parallel in vitro and in planta screening increases the chances to isolate PGPMs

As an important step following the classical characterization of the isolates via various in vitro assays, we established an optimized screening method that enables direct *in planta* testing of their efficiency on tree seedlings, specifically *Picea abies* (spruce), under both drought and well-watered conditions. The drought conditions applied in this system were severe, as indicated by a decrease in seedling survival from over 75% (WW) to approximately 50% or less under drought (Table S9).

The efficacy of inoculation with bacteria depends on their rhizosphere competence for the specific host plant [110, 121]. 27 of 29 bacteria were successfully re-isolated from spruce roots after 3 weeks, demonstrating their rhizosphere competence and persistence at the root level. Only *Psychrobacillus* sp. KF94 and *Viridibacillus* sp. KF108 could not be re-isolated. The fact that their inoculation nevertheless increased plant survival suggests that they had a positive effect at least initially, but were unable to establish themselves at the roots. This is not a new phenomenon. For example, *Azospirillum lipoferum* stimulated root growth in maize but could not be detected by quantitative PCR around 4 weeks after inoculation [122].

Finally, of the 86% of isolates that exhibited PGP to several degrees, 2 out of 29 tested isolates supported seedling growth under drought. This underscores the limitations of in vitro screenings for identifying PGPMs, particularly in translating results from artificial conditions to more realistic field-like environments [37, 110, 123–125], and the importance of suitable in vivo experiments. The fact that in vitro screening results are not directly transferrable to more natural in vivo conditions was true also for our study, in which all strains with beneficial effects in vivo exhibited only moderately high scores in the in vitro assays, ranging from 10 to 13 (except for *Paraburkholderia* sp. Ke296, which achieved the highest recorded score of 18). In vivo experiments under more natural conditions are therefore essential for validating the efficacy of PGPMs before proceeding with time-intensive field or greenhouse trials [126]. The optimized 24-deep-well plate-based system was developed to bridge this gap between in vitro screenings and field application, enabling a controlled and rapid screening of promising isolates for their beneficial impact on seedling fitness. In this approach, bacteria were applied directly to the target plant (spruce), avoiding the use of common model plants such as *Arabidopsis* or tomato, often used as proxies.

The *in planta* screening revealed that two strains, *Caballeronia* sp. Ke431 and *Paraburkholderia* sp. Ke296, were able to promote spruce growth under DS, making them particularly noteworthy. As mentioned before, plants are often able to maintain their above-ground growth under moderate drought. Severe drought, on the contrary, leads to a decrease in shoot growth, whereas the roots continue to grow [21]. This was also observed in the present study for spruce seedlings under drought, as control plants under DS sometimes showed an increased root length compared to control plants under WW conditions (Figure S4 B), although they had a reduced survival rate (Table S9). After inoculation with *Caballeronia* sp. Ke431 and *Paraburkholderia* sp. Ke296 under DS, this effect was especially pronounced (Figure S4). This would enable seedlings to reach deeper soil layers where more water is present while the upper soil layers are more affected by drought [15, 44]. Incidentally, both DS-alleviating strains were able to fix nitrogen, for which there is evidence that this trait is involved in drought resistance mechanisms in seedlings: In *P. abies* for example, organic N-supply improved survival after planting in the field [14].

Additionally, *Caballeronia* sp. Ke431 as well as five other strains promoted spruce growth under WW conditions: *Herbaspirillum* sp. KF115, *Streptomyces* sp. KF215 and three *Paraburkholderia* strains (Ke15, Ke24, Ke162). Generally, root fresh weight was particularly positively affected by inoculation with the bacteria tested in this study. It is well known that PGPB present in the rhizosphere or endosphere that are able to convert plant-produced tryptophan to IAA, can significantly promote plant growth [49]. Interestingly, IAA and ACC deaminase production were exhibited by six of the seven most-promising PGPB (Table S11), suggesting that these traits may be particularly important for PGP. All strains tolerated pH values between 4 and 5, being a prerequisite for surviving the relatively acidic pH of 5 in forest soils. Siderophore production and nitrogen fixation were the least common PGP traits, observed in only two strains each. The four *Paraburkholderia* strains shared most of their PGP abilities, indicating that some PGP traits might be genus-specific (Table S11).

Even though inoculation with *Psychrobacillus* sp. KF94 and *Viridibacillus* sp. KF108—both representing genera for which PGP potential is so far scarcely documented—improved survival under drought conditions, they did not promote plant growth. Other strains from genera with little-known PGP activity, such as *Collimonas* sp. Ke374, showed no positive effects *in planta* but strong in vitro performance, including the production of IAA, siderophores, ACC deaminase, and phosphate solubilization. This discrepancy underlines the complexity of plant–microbe interactions in natural systems and suggests that additional factors beyond those tested in classical in vitro assays are essential for effective PGP under environmental conditions. The results also suggest that genera for which PGP potential is still poorly understood may confer other, less well-characterized benefits (such as improved drought survival), or that their functions are highly context-dependent and require further investigation *in planta*. Moreover, factors such as inoculum dose and application frequency can influence these outcomes [127]. However, for all four PGP genera identified within the 24-well-based test system, strains have been isolated in previous studies, which were demonstrated to be plant beneficial. The genus *Herbaspirillum* has not been tested on spruce so far, but demonstrated PGP effects under DS for example in maize, where inoculation with *Herbaspirillum seropedicae* ZAE94 increased yield by up to 34% [128]. An inoculation of hybrid white spruce with six bacteria, among them *Caballeronia sordidicola* LS-Sr, enhanced seedling growth by accumulating 607% more biomass in comparison to the control plants [49]. *Paraburkholderia phytofirmans* LP-R1r, *Caballeronia sordidicola* HP-S1r and *Caballeronia udeis* LP-R2r significantly increased spruce and pine seedling length by up to 60% and seedling biomass by up to 302% [129]. The plant beneficial *Streptomyces* AcH505 strain promoted root branching and lateral root formation in Norway spruce [53, 54]. However, these examples only address spruce growth promotion under well-watered conditions. To our knowledge, this is the first report of PGPB improving spruce growth under drought.

Overall, the 24-well in vivo screening approach developed in this study proved particularly useful due to its relatively high throughput capacity. This prevented losing potential PGPB, which did not exhibit strong effects within the in vitro assays but showed their beneficial properties only when being applied to plants. At the same time, it verifies that potential PGP strains also exhibit their traits on the target plant and not only in an artificial assay or a model plant.

Taken together, PGPMs offer promising and sustainable options for ecosystem restoration and forestry and the majority of soil microbes play important roles in supporting plant survival under stressful conditions [34]. However, careful consideration of their ecological safety remains essential. The introduction of isolated strains into forest ecosystems should take into account potential unintended consequences, such as impacts on native microbial communities or non-target organisms, highlighting the importance of thorough risk assessment and ongoing monitoring within restoration initiatives [130].

## Conclusion

This study constitutes one of the first larger scale screenings for PGPM isolated from forest soils with axenic application to tree seedlings under drought stress. The double screening in vitro and *in planta* has proven beneficial in identifying PGP isolates that would have been overlooked based on the in vitro scoring alone. Overall, three microbes can be especially highlighted: *Streptomyces* sp. Ke434 due to its high stress resistance, as well as *Caballeronia* sp. Ke431 and *Paraburkholderia* sp. Ke296 which showed positive effects on spruce growth under drought conditions. *Paraburkholderia* sp. Ke296 also exhibited the most PGP traits of all tested strains. The *in planta* screening system offers a range of additional application possibilities, including adaptation to other plant species or testing further stress conditions such as heat, cold and salt stress. Additionally, the system could be adapted for fungal inoculations or simultaneous inoculations together with bacteria.

## Supplementary Information

Supplementary Material 1.

## Data Availability

The datasets generated or analyzed during the current study are included in this article and its supplementary information files or are available from the corresponding author upon reasonable request. The raw sequencing data have been deposited in the NCBI Sequence Read Archive (SRA) under the following accession numbers: SRR33458690-SRR33458820 for bacteria and SRR33459453-SRR33459578 for fungi (BioProject: PRJNA1259936). The NCBI GenBank accession numbers for the bacterial isolates are PV918716-PV918751, and for the fungal isolates PV918686-PV918701.
