# Supplementary material for: Microbial allies against drought stress: an optimized screening method to improve seedling survival for forest restorations
Source: Environ Microbiome. 2026 Mar 25;21:45. doi: 10.1186/s40793-026-00878-8 (PMC13023185; doi:10.1186/s40793-026-00878-8)
Supplement: Supplementary file 1 — Supplementary Material 1. [file 40793_2026_878_MOESM1_ESM.docx]

**Supplementary material**

Table S1: Precipitation [mm] in the week before sampling at the two different sites.

| **Freising (Kranzberg)** | | **Kelheim** | |
| --- | --- | --- | --- |
| **Date** | **Precipitation [mm]** | **Date** | **Precipitation [mm]** |
| 07/21/2021 | 0 | 09/01/2021 | 0 |
| 07/22/2021 | 0 | 09/02/2021 | 0 |
| 07/23/2021 | 0 | 09/03/2021 | 0 |
| 07/24/2021 | 5 | 09/04/2021 | 0 |
| 07/25/2021 | 5.5 | 09/05/2021 | 0 |
| 07/26/2021 | 8.7 | 09/06/2021 | 0 |
| 07/27/2021 | 1 | 09/07/2021 | 0 |

Table S2: Bacterial strains utilized in this study including their isolation medium and origin.

| **Strain** | **Isolation medium** | **Origin** | **Accession No.** |
| --- | --- | --- | --- |
| *Pseudomonas* sp. Ke1 | M9 medium | Beech roots in Kelheim | PV918716 |
| *Paraburkholderia* sp. Ke15 | M9 medium | Beech roots in Kelheim | PV918717 |
| *Paraburkholderia* sp. Ke24 | M9 medium | Beech roots in Kelheim | PV918718 |
| *Rhizobium* sp. Ke26 | M9 medium | Beech roots in Kelheim | PV918719 |
| *Caballeronia* sp. Ke41 | M9 medium | Beech roots in Kelheim | PV918720 |
| *Caballeronia* sp. Ke57 | M9 medium | Spruce roots in Kelheim | PV918721 |
| *Bacillus* sp. Ke157 | King’s B medium | Beech roots in Kelheim | PV918722 |
| *Paraburkholderia* sp. Ke162 | King’s B medium | Beech roots in Kelheim | PV918723 |
| *Paraburkholderia* sp. Ke296 | MMNC medium | Beech roots in Kelheim | PV918724 |
| *Paraburkholderia* sp. Ke341 | MMNC medium | Beech roots in Kelheim | PV918725 |
| *Collimonas* sp. Ke374 | MMNC medium | Spruce roots in Kelheim | PV918726 |
| *Paraburkholderia* sp. Ke398 | MMNC medium | Spruce roots in Kelheim | PV918727 |
| *Caballeronia* sp. Ke431 | MMNC medium | Spruce roots in Kelheim | PV918728 |
| *Streptomyces* sp. Ke434 | KM4 agar | Beech roots in Kelheim | PV918729 |
| *Rhodococcus* sp. Ke442 | KM4 agar | Beech roots in Kelheim | PV918730 |
| *Streptomyces* sp. Ke462 | KM4 agar | Beech roots in Kelheim | PV918731 |
| *Rhodococcus* sp. Ke466 | KM4 agar | Beech roots in Kelheim | PV918732 |
| *Sporosarcina* sp. Ke477 | KM4 agar | Beech roots in Kelheim | PV918733 |
| *Streptomyces* sp. Ke502 | KM4 agar | Spruce roots in Kelheim | PV918734 |
| *Streptomyces* sp. Ke507 | KM4 agar | Spruce roots in Kelheim | PV918735 |
| *Bacillus* sp. KF3 | King’s B medium | Beech roots in Kranzberg | PV918736 |
| *Lysinibacillus* sp. KF19 | King’s B medium | Beech roots in Kranzberg | PV918737 |
| *Bacillus* sp. KF21 | King’s B medium | Beech roots in Kranzberg | PV918738 |
| *Bacillus* sp. KF47 | MMNC medium | Spruce roots in Kranzberg | PV918739 |
| *Pseudomonas* sp. KF80 | King’s B medium | Beech roots in Kranzberg | PV918740 |
| *Psychrobacillus* sp. KF94 | KM4 agar | Spruce roots in Kranzberg | PV918741 |
| *Viridibacillus* sp. KF108 | MMNC medium | Spruce roots in Kranzberg | PV918742 |
| *Herbaspirillum* sp. KF115 | MMNC medium | Spruce roots in Kranzberg | PV918743 |
| *Caballeronia* sp. KF127 | MMNC medium | Beech roots in Kranzberg | PV918744 |
| *Streptomyces* sp. KF143 | King’s B medium | Beech roots in Kranzberg | PV918745 |
| *Streptomyces* sp. KF147 | MMNC medium | Spruce roots in Kranzberg | PV918746 |
| *Pseudomonas* sp. KF164 | M9 medium | Beech roots in Kranzberg | PV918747 |
| *Streptomyces* sp. KF207 | KM4 agar | Spruce roots in Kranzberg | PV918748 |
| *Streptomyces* sp. KF209 | MMNC medium | Spruce roots in Kranzberg | PV918749 |
| *Streptomyces* sp. KF215 | KM4 agar | Spruce roots in Kranzberg | PV918750 |
| *Variovorax* sp. KF227 | M9 medium | Spruce roots in Kranzberg | PV918751 |

Table S3: Fungal strains utilized in this study including their isolation medium and origin.

| **Strain** | **Isolation medium** | **Origin** | **Accession No.** |
| --- | --- | --- | --- |
| *Umbelopsis* sp. F4 | MMNC medium | Beech roots in Kelheim | PV918698 |
| *Tolypocladium* sp. F5 | MMNC medium | Spruce roots in Kelheim | PV918699 |
| *Metapochonia* sp. F6 | M9 medium | Spruce roots in Kelheim | PV918700 |
| *Umbelopsis* sp. F10 | MMNC medium | Spruce roots in Kelheim | PV918686 |
| *Umbelopsis* sp. F11 | MMNC medium | Spruce roots in Kelheim | PV918694 |
| *Umbelopsis* sp. F12 | MMNC medium | Spruce roots in Kelheim | PV918687 |
| *Umbelopsis* sp. F14 | MMNC medium | Spruce roots in Kelheim | PV918688 |
| *Lycoperdon* sp. F31 | MMNC medium | Fruiting body near beech roots in Kelheim | - |
| *Collybiopsis* sp. F32 | MMNC medium | Fruiting body near spruce roots in Kelheim | PV918690 |
| *Umbelopsis* sp. F34 | MMNC medium | Spruce roots in Kelheim | PV918691 |
| *Tolypocladium* sp. F36 | MMNC medium | Spruce roots in Kelheim | PV918692 |
| *Podila* sp. F37 | MMNC medium | Spruce roots in Kelheim | PV918693 |
| *Podila* sp. F40 | King’s B medium | Spruce roots in Kelheim | PV918697 |
| *Umbelopsis* sp. F7 | MMNC medium | Beech roots in Kranzberg | PV918701 |
| *Umbelopsis* sp. F17 | MMNC medium | Beech roots in Kranzberg | PV918689 |
| *Mortierella* sp. F38 | MMNC medium | Beech roots in Kranzberg | PV918695 |
| *Mortierella* sp. F39 | MMNC medium | Spruce roots in Kranzberg | PV918696 |

Table S4: PEG, NaCl and H⁺ concentrations used in the stress tolerance assays, along with the defined thresholds for assigning scores from 0 to 3 for both stress tolerance and IAA production assays.

| **PEG [MPa]** | **NaCl [%]** | **pH (H^+^)** | **Bacterial IAA production [µg mL^‑1^]** | **Fungal IAA production fungi [µg mg^-1^]** | **Scoring** |
| --- | --- | --- | --- | --- | --- |
| 0 | 0 | 7 and 8 | <5 | <0.5 | 0 |
| -0.25 | 0.1 | 6 | 5-10 | 0.5-1 | 1 |
| -0.5 | 2.5 | - |  |  | 1 |
| -0.75 | 3.5 | 5 | 10-15 | 1-5 | 2 |
| -1.0 | 5.5 | - |  |  | 2 |
| -1.25 | 7.5 | 4 | >15 | >5 | 3 |
| -1.5 | 12 | 3 |  |  | 3 |
| -1.75 | 15 | 2 |  |  | 3 |

Table S5: Fungal phyla and genera isolated as single strains from beech and spruce roots in Kelheim and Kranzberg.

| **Phylum** | **Genus** | **Number of isolates** | | | | |
| --- | --- | --- | --- | --- | --- | --- |
|  |  | **Kranzberg** | | **Kelheim** | | **Sum** |
|  |  | **Beech** | **Spruce** | **Beech** | **Spruce** |  |
| Ascomycota | *Penicillium* | 4 | 2 | 2 | 0 | 8 |
|  | *Cladosporium* | 0 | 3 | 0 | 0 | 3 |
|  | *Geomyces*/*Pseudogymnoascus* | 0 | 0 | 0 | 1 | 1 |
|  | *Trichoderma* | 0 | 0 | 3 | 0 | 3 |
|  | *Tolypocladium* | 0 | 0 | 0 | 2 | 2 |
|  | *Acremonium*/*Scopulariopsis* | 0 | 0 | 1 | 1 | 2 |
|  | *Verticillium*/*Simplicillium* | 0 | 0 | 2 | 0 | 2 |
|  | *Metapochonia* | 0 | 0 | 0 | 1 | 1 |
| Mucoromycota | *Umbelopsis* | 2 | 0 | 1 | 5 | 8 |
|  | *Mortierella* | 1 | 2 | 0 | 1 | 4 |
|  | *Podila* | 0 | 0 | 0 | 2 | 2 |
|  | *Mucor* | 0 | 0 | 1 | 0 | 1 |
| Basidiomycota | *Lycoperdon* | 0 | 0 | 1 | 0 | 1 |
|  | *Collybiopsis* | 0 | 0 | 0 | 1 | 1 |
| Sum | | 7 | 7 | 11 | 14 | 39 |

Table S6: Recovery of bacteria from the 2-3 highest NaCl, PEG and H^+^ concentrations. The highest concentration from which strains could recover is indicated. Non-recovery of strains is indicated by “-“.

| **Strain** | **NaCl** | **PEG** | **H^+^** |
| --- | --- | --- | --- |
| *Pseudomonas* sp. Ke1 | - | -1.25 | - |
| *Paraburkholderia* sp. Ke15 | - | -1.5 | 4 |
| *Paraburkholderia* sp. Ke24 | - | - | 4 |
| *Rhizobium* sp. Ke26 | - | - | 4 |
| *Caballeronia* sp. Ke41 | - | - | 4 |
| *Caballeronia* sp. Ke57 | 15 | -1.75 | 3 |
| *Bacillus* sp. Ke157 | 15 | -1.75 | 2 |
| *Paraburkholderia* sp. Ke162 | - | - | 4 |
| *Paraburkholderia* sp. Ke296 | 15 | -1.25 | 4 |
| *Paraburkholderia* sp. Ke341 | 15 | -1.75 | 4 |
| *Collimonas* sp. Ke374 | 12 | -1.75 | 4 |
| *Paraburkholderia* sp. Ke398 | 15 | -1.75 | 4 |
| *Caballeronia* sp. Ke431 | - | - | 4 |
| *Streptomyces* sp. Ke434 | 15 | -1.75 | 3 |
| *Rhodococcus* sp. Ke442 | 15 | -1.75 | 3 |
| *Streptomyces* sp. Ke462 | 15 | -1.75 | 4 |
| *Rhodococcus* sp. Ke466 | 15 | -1.5 | 2 |
| *Sporosarcina* sp. Ke477 | 15 | -1.75 | - |
| *Streptomyces* sp. Ke502 | 15 | -1.75 | 3 |
| *Streptomyces* sp. Ke507 | 15 | -1.75 | 3 |
| *Bacillus* sp. KF3 | 15 | -1.75 | 3 |
| *Lysinibacillus* sp. KF19 | 15 | -1.75 | - |
| *Bacillus* sp. KF21 | 15 | -1.75 | 4 |
| *Bacillus* sp. KF47 | 15 | -1.75 | 3 |
| *Pseudomonas* sp. KF80 | - | - | - |
| *Psychrobacillus* sp. KF94 | - | -1.75 | 4 |
| *Viridibacillus* sp. KF108 | 15 | -1.75 | 2 |
| *Herbaspirillum* sp. KF115 | - | - | - |
| *Caballeronia* sp. KF127 | - | - | - |
| *Streptomyces* sp. KF143 | 15 | -1.75 | - |
| *Streptomyces* sp. KF147 | 15 | -1.75 | - |
| *Pseudomonas* sp. KF164 | - | - | - |
| *Streptomyces* sp. KF207 | 12 | -1.75 | 4 |
| *Streptomyces* sp. KF209 | 12 | -1.75 | 4 |
| *Streptomyces* sp. KF215 | 15 | -1.75 | - |
| *Variovorax* sp. KF227 | - | - | - |

Table S7: Bacterial IAA production with and without tryptophan in µg mL^‑1^ with the respective standard deviation. PC = positive control.

| **Strain** | **IAA production [µg mL^-1^]** | |
| --- | --- | --- |
|  | **With tryptophan** | **Without tryptophan** |
| *Pseudomonas* sp. Ke1 | 25.4 ± 7.0 | 6.5 ± 0.3 |
| *Paraburkholderia* sp. Ke15 | 7.8 ± 1.9 | 6.2 ± 2.7 |
| *Paraburkholderia* sp. Ke24 | 9.8 ± 3.8 | 8.2 ± 5.6 |
| *Rhizobium* sp. Ke26 | 10.6 ± 2.1 | 9.9 ± 0.8 |
| *Caballeronia* sp. Ke41 | 8.3 ± 5.9 | 2.3 ± 1.5 |
| *Caballeronia* sp. Ke57 | 10.4 ± 1.0 | 9.2 ± 6.8 |
| *Bacillus* sp. Ke157 | 10.9 ± 1.0 | 1.1 ± 0.8 |
| *Paraburkholderia* sp. Ke162 | 5.7 ± 1.4 | 6.0 ± 3.4 |
| *Paraburkholderia* sp. Ke296 | 2.7 ± 1.2 | 1.8 ± 1.2 |
| *Paraburkholderia* sp. Ke341 | 7.7 ± 2.9 | 9.6 ± 7.0 |
| *Collimonas* sp. Ke374 | 10.1 ± 3.0 | 5.3 ± 2.2 |
| *Paraburkholderia* sp. Ke398 | 2.3 ± 1.2 | 1.4 ± 0.4 |
| *Caballeronia* sp. Ke431 | 10.0 ± 1.0 | 5.8 ± 3.2 |
| *Streptomyces* sp. Ke434 | 1.4 ± 0.6 | 0.4 ± 0.1 |
| *Rhodococcus* sp. Ke442 | 0.5 ± 0.4 | 0.4 ± 0.6 |
| *Streptomyces* sp. Ke462 | 7.7 ± 0.7 | 3.6 ± 2.4 |
| *Rhodococcus* sp. Ke466 | 1.9 ± 0.2 | 0.1 ± 0.1 |
| *Sporosarcina* sp. Ke477 | 18.0 ± 7.8 | 6.6 ± 3.0 |
| *Streptomyces* sp. Ke502 | 2.2 ± 0.3 | 3.2 ± 0.2 |
| *Streptomyces* sp. Ke507 | 1.2 ± 0.0 | 0.3 ± 0.1 |
| *Bacillus* sp. KF3 | 11.0 ± 3.1 | 2.3 ± 0.6 |
| *Lysinibacillus* sp. KF19 | 41.1 ± 12.3 | 2.7 ± 1.5 |
| *Bacillus* sp. KF21 | 6.1 ± 1.1 | 1.6 ± 0.5 |
| *Bacillus* sp. KF47 | 8.6 ± 3.5 | 2.3 ± 1.1 |
| *Pseudomonas* sp. KF80 | 24.4 ± 4.6 | 8.4 ± 1.4 |
| *Psychrobacillus* sp. KF94 | 19.9 ± 11.6 | 10.0 ± 12.6 |
| *Viridibacillus* sp. KF108 | 55.7 ± 11.8 | 14.2 ± 9.2 |
| *Herbaspirillum* sp. KF115 | 11.0 ± 2.1 | 4.6 ± 1.1 |
| *Caballeronia* sp. KF127 | 39.4 ± 2.7 | 7.5 ± 3.1 |
| *Streptomyces* sp. KF143 | 11.6 ± 1.9 | 2.8 ± 2.4 |
| *Streptomyces* sp. KF147 | 19.9 ± 12.1 | 8.4 ± 13.6 |
| *Pseudomonas* sp. KF164 | 39.4 ± 3.4 | 4.9 ± 1.8 |
| *Streptomyces* sp. KF207 | 20.7 ± 3.0 | 20.3 ± 2.4 |
| *Streptomyces* sp. KF209 | 1.1 ± 0.7 | 1.6 ± 0.9 |
| *Streptomyces* sp. KF215 | 16.6 ± 8.7 | 7.0 ± 8.7 |
| *Variovorax* sp. KF227 | 1.0 ± 0.1 | 1.7 ± 0.2 |
| *Herbaspirillum frisingense* GSF30 (PC) | 12.4 ± 2.1 | 8.5 ± 1.0 |

Table S8: Fungal IAA production with and without tryptophan in µg mg^‑1^ with the respective standard deviation.

| **Strain** | **IAA production [µg mg^-1^]** | |
| --- | --- | --- |
|  | **With tryptophan** | **Without tryptophan** |
| *Umbelopsis* sp. F4 | 0.36 ± 0.15 | 0.04 ± 0.02 |
| *Tolypocladium* sp. F5 | 0.50 ± 0.20 | 0.02 ± 0.02 |
| *Metapochonia* sp. F6 | 0.05 ± 0.03 | 0.02 ± 0.03 |
| *Umbelopsis* sp. F7 | 0.59 ± 0.38 | 0.03 ± 0.02 |
| *Umbelopsis* sp. F10 | 0.89 ± 0.08 | 0.02 ± 0.00 |
| *Umbelopsis* sp. F11 | 5.53 ± 2.25 | 0.13 ± 0.05 |
| *Umbelopsis* sp. F12 | 0.43 ± 0.15 | 0.00 ± 0.01 |
| *Umbelopsis* sp. F14 | 1.75 ± 0.42 | 0.01 ± 0.00 |
| *Umbelopsis* sp. F17 | 0.82 ± 0.34 | -0.02 ± 0.06 |
| *Lycoperdon* sp. F31 | 1.74 ± 0.09 | 0.00 ± 0.01 |
| *Collybiopsis* sp. F32 | 1.78 ± 1.07 | -0.05 ± 0.01 |
| *Umbelopsis* sp. F34 | 0.90 ± 0.27 | -0.02 ± 0.02 |
| *Tolypocladium* sp. F36 | 1.41 ± 0.23 | 0.04 ± 0.04 |
| *Podila* sp. sp. F37 | 2.21 ± 0.37 | 0.01 ± 0.01 |
| *Mortierella* sp. F38 | 4.73 ± 1.71 | -0.02 ± 0.04 |
| *Mortierella* sp. F39 | 6.35 ± 1.46 | -0.08 ± 0.06 |
| *Podila* sp. F40 | 3.99 ± 1.29 | -0.03 ± 0.03 |

Table S9: Summary of the plant survival rates and colony forming units (CFUs) after inoculation with the different isolates and growth for 3 weeks in a phytochamber. Survival rates are indicated in relation to the respective uninoculated control plants of each experiment as relative values. An improvement of the survival rate is highlighted in grey.

| **Strain** | **Increase/decrease in survival (fold change, relative values)** | | **CFU count** | |
| --- | --- | --- | --- | --- |
|  | **WW** | **DS** | **WW** | **DS** |
| *Pseudomonas* sp. Ke1 | 1.47 | 2.0 | 1.5 x 10^6^ | 2.6 x 10^5^ |
| *Paraburkholderia* sp. Ke15 | 1.35 | 0.75 | 3.1 x 10^7^ | 2.5 x 10^7^ |
| *Paraburkholderia* sp. Ke24 | 1.18 | 0.75 | 4.9 x 10^7^ | 1.5 x 10^7^ |
| *Caballeronia* sp. Ke41 | 0.8 | 0.89 | 1.5 x 10^7^ | 7.5 x 10^6^ |
| *Caballeronia* sp. Ke57 | 0.94 | 0.71 | 6.8 x 10^6^ | 3.4 x 10^5^ |
| *Paraburkholderia* sp. Ke162 | 0.91 | 0.72 | 1.3 x 10^7^ | 1.8 x 10^7^ |
| *Paraburkholderia* sp. Ke296 | 1.11 | 1.18 | 5.7 x 10^6^ | 4.8 x 10^6^ |
| *Paraburkholderia* sp. Ke341 | 0.83 | 0.87 | 3.9 x 10^7^ | 3.5 x 10^7^ |
| *Collimonas* sp. Ke374 | 1.0 | 1.07 | 3.0 x 10^7^ | 1.0 x 10^7^ |
| *Paraburkholderia* sp. Ke398 | 0.83 | 0.63 | 2.7 x 10^7^ | 7.7 x 10^7^ |
| *Caballeronia* sp. Ke431 | 1.0 | 3.01 | 6.2 x 10^5^ | 2.3 x 10^5^ |
| *Streptomyces* sp. Ke434 | 1.11 | 1.0 | 1.8 x 10^6^ | 5.0 x 10^6^ |
| *Rhodococcus* sp. Ke442 | 0.96 | 0.72 | 1.3 x 10^6^ | 1.8 x 10^6^ |
| *Streptomyces* sp. Ke462 | 0.83 | 1.0 | 1.6 x 10^4^ | 8.7 x 10^5^ |
| *Rhodococcus* sp. Ke466 | 0.94 | 0.5 | 2.1 x 10^6^ | 4.0 x 10^6^ |
| *Sporosarcina* sp. Ke477 | 0.72 | 1.36 | 2.8 x 10^6^ | 1.2 x 10^4^ |
| *Streptomyces* sp. Ke507 | 0.61 | 0 | 2.5 x 10^5^ | dead |
| *Bacillus* sp. KF3 | 1.1 | 2.0 | 4.8 x 10^6^ | 1.6 x 10^4^ |
| *Bacillus* sp. KF21 | 1.0 | 0.25 | 9.3 x 10^4^ | 7.4 x 10^5^ |
| *Bacillus* sp. KF47 | 1.1 | 1.78 | 2.5 x 10^4^ | 1.4 x 10^4^ |
| *Psychrobacillus* sp. KF94 | 1.2 | 2.83 | - | - |
| *Viridibacillus* sp. KF108 | 1.47 | 3.16 | - | 4.9 x 10^3^ |
| *Herbaspirillum* sp. KF115 | 1.17 | 1.57 | 1.5 x 10^7^ | 2.9 x 10^6^ |
| *Caballeronia* sp. KF127 | 0.78 | 1.36 | 3.6 x 10^7^ | 7.0 x 10^6^ |
| *Streptomyces* sp. KF143 | 0.83 | 0.63 | 6.6 x 10^6^ | 4.8 x 10^6^ |
| *Streptomyces* sp. KF147 | 1.47 | 1.0 | 8.9 x 10^5^ | 3.1 x 10^6^ |
| *Pseudomonas* sp. KF164 | 0.91 | 1.14 | 1.3 x 10^6^ | 9.6 x 10^5^ |
| *Streptomyces* sp. KF207 | 0.8 | 0.67 | 1.5 x 10^5^ | 3.6 x 10^5^ |
| *Streptomyces* sp. KF215 | 1.0 | 1.18 | 4.0 x 10^5^ | 1.9 x 10^6^ |

Table S10: Summary of the effects of the isolates on plant growth promotion in comparison their respective control in the 24-well test system. Significant plant growth promotion after inoculation is marked with “+”. In the experiment with Ke431 and Ke434 inoculation, dry weight measurement was not performed for the control plants (n.c.). Bacteria are listed in descending order of importance with respect to their ability to promote plant growth.

| **Inoculated bacteria** | **Significant plant growth promotion, indicated by “+”** | | | | | | | | | | | | | |
| --- | --- | --- | --- | --- | --- | --- | --- | --- | --- | --- | --- | --- | --- | --- |
| **Strain** | **Seedling length** | | **Seedling fresh weight** | | **Root length** | | **Root fresh weight** | | **Shoot length** | | **Shoot fresh weight** | | **Seedling dry weight** | |
|  | **WW** | **DS** | **WW** | **DS** | **WW** | **DS** | **WW** | **DS** | **WW** | **DS** | **WW** | **DS** | **WW** | **DS** |
| *Caballeronia* sp. Ke431 | **+** | **+** | **+** |  | **+** | **+** | **+** |  |  |  | **+** |  | n.c. | |
| *Paraburkholderia* sp. Ke296 |  | **+** |  |  |  | **+** |  |  |  |  |  |  |  |  |
| *Paraburkholderia* sp. Ke15 | **+** |  | **+** |  | **+** |  | **+** |  | **+** |  | **+** |  | **+** |  |
| *Paraburkholderia* sp. Ke24 | **+** |  | **+** |  | **+** |  | **+** |  |  |  | **+** |  | **+** |  |
| *Herbaspirillum* sp. KF115 |  |  | **+** |  |  |  | **+** |  |  |  | **+** |  | **+** |  |
| *Streptomyces* sp. KF215 | **+** |  |  |  | **+** |  | **+** |  |  |  |  |  |  |  |
| *Paraburkholderia* sp. Ke162 |  |  |  |  |  |  | **+** |  |  |  |  |  |  |  |
| *Psychrobacillus* sp. KF94 |  |  |  |  |  |  |  |  |  |  |  |  |  | **+** |
| *Rhodococcus* sp. Ke466 |  |  |  |  |  |  |  |  |  |  |  |  | **+** |  |
| *Bacillus* sp. KF21 |  |  |  |  |  |  |  |  |  |  |  |  | **+** |  |
| No effect *in vivo: Pseudomonas* sp. Ke1, *Caballeronia* sp. Ke41, *Caballeronia* sp. Ke57, *Paraburkholderia* sp. Ke431, *Collimonas* sp. Ke374, *Paraburkholderia* sp. Ke398, *Streptomyces* sp. Ke434, *Rhodococcus* sp. Ke442, *Streptomyces* sp. Ke462, *Sporosarcina* sp. Ke477, *Streptomyces*/*Kitasatospora* sp. Ke507, *Bacillus* sp. KF3, *Bacillus* sp. KF47, *Viridibacillus* sp. KF108, *Caballeronia* sp. KF127, *Streptomyces* sp. KF143, *Streptomyces* sp. KF147, *Pseudomonas* sp. KF164, *Streptomyces* sp. KF207 | | | | | | | | | | | | | | |

Table S11: Summary of the PGP properties and stress tolerance of the seven PGPB identified within the 24-well-based test system. The assigned scores for PEG, NaCl, and pH tolerance, as well as for IAA-production, are indicated. ACC-utilization, N-fixation, P-solubilization and siderophore (S) production is marked with “X”.

| **Strain** | **PEG** | **NaCl** | **pH** | **IAA** | **ACC** | **N** | **P** | **S** |
| --- | --- | --- | --- | --- | --- | --- | --- | --- |
| *Paraburkholderia* sp. Ke15 | 2 | 1 | 3 | 1 | X | - | X | - |
| *Paraburkholderia* sp. Ke24 | 2 | 1 | 3 | 1 | X | - | X | - |
| *Paraburkholderia* sp. Ke162 | 2 | 1 | 3 | 1 | X | - | X | X |
| *Paraburkholderia* sp. Ke296 | 2 | 1 | 3 | - | X | X | X | - |
| *Caballeronia* sp. Ke431 | 1 | 1 | 2 | 2 | X | X | - | - |
| *Herbaspirillum* sp. KF115 | 1 | 1 | 2 | 2 | X | - | - | X |
| *Streptomyces* sp. KF215 | 3 | 2 | 2 | 3 | - | - | - | - |

A

C

E

B

D

F

Figure S1: Comparison of the initially used internal controls under well-watered conditions. CAsum is the average of the internal controls CA1 and CA2. CBsum is the average of the internal controls CB1-CB4. There is no significant difference between the average of the internal controls and the internal controls.

Figure S2: Relative abundance [%] of the fungal phyla in beech and spruce rhizosphere communities in Kelheim and Kranzberg. Community composition was evaluated using the microbiome packages (v1.24.0, Leo et al. 2017) in R. Bray-Curtis distance was calculated using the R package vegan (v2.6.6, Oksanen et al. 2017). The plot was generated using origin pro (version 2021b).


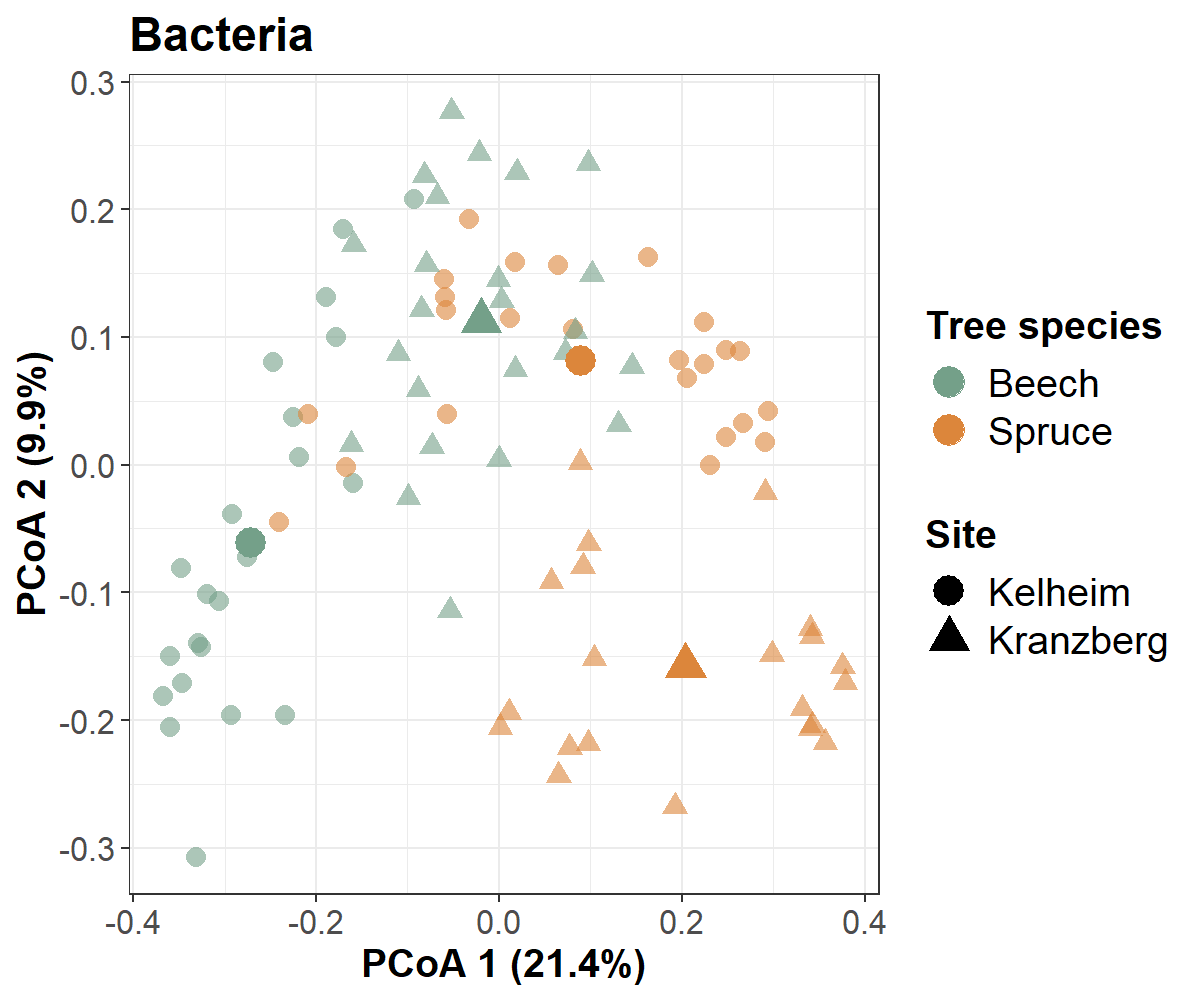

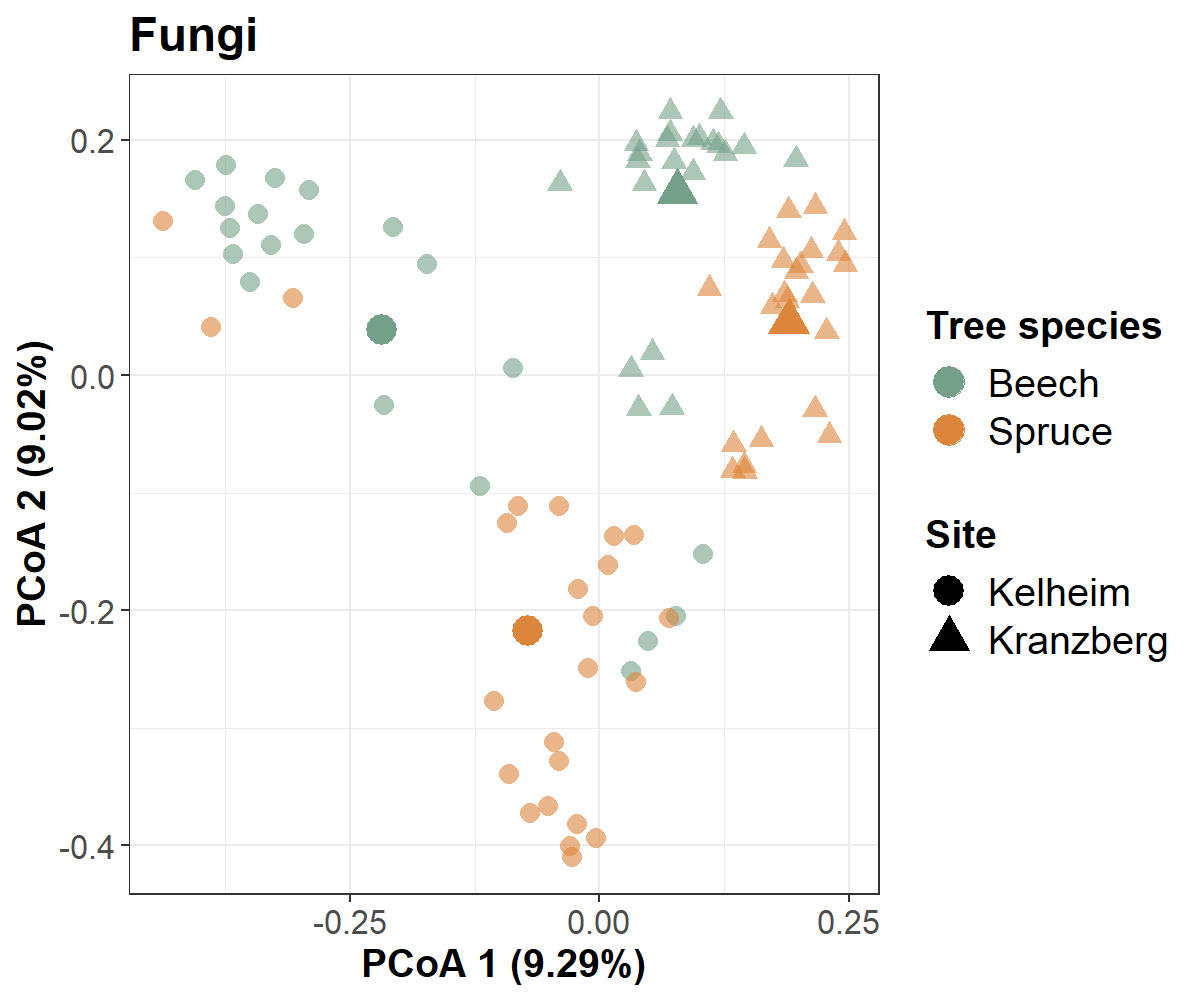


B

A

Figure S3: Principal Coordinate Analysis (PCoA) showing the site- and tree species-related differences in the (A) bacterial and (B) fungal rhizosphere community composition. The average of each combination is highlighted by a more intense color and a bigger size of the respective symbols.

A

B

Figure S4: Root length after inoculation with (A) Caballeronia sp. Ke431 and (B) Paraburkholderia sp. Ke296 and Streptomyces sp. KF215.

A

B

C


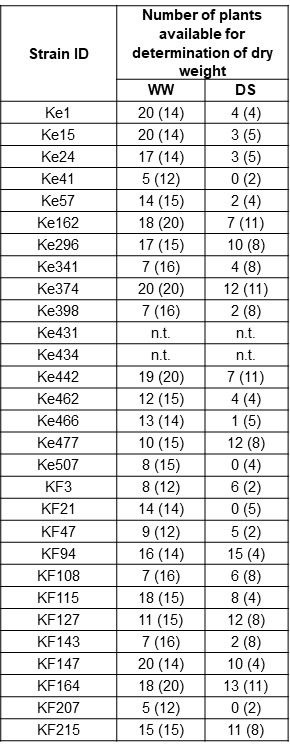


Figure S5: Absolute changes in seedling dry weight under (A) well-watered (WW) and (B) drought stress (DS) conditions after inoculation with individual bacterial isolates. The red zero baseline represents the mean of the uninoculated control within each experiment. Positive values indicate an absolute increase, and negative values a decrease in dry weight compared to the respective control. Individual experiments are separated by dashed lines. Significant plant growth-promotion after inoculation with the isolates compared to the respective control is marked with asterisks. (C) The number of plants available for dry weight determination depended on the survival rate and excluded plants used for CFU count. The number of control plants is given in brackets.

Figure S6: Absolute increase or decrease in root length under well-watered conditions after inoculation with individual bacterial isolates. The red zero baseline represents the mean of the uninoculated control within each experiment. Positive values indicate an absolute increase, and negative values a decrease in length compared to the respective control. Individual experiments are separated by dashed lines. Significant plant growth-promotion after inoculation with the isolates compared to the respective control is marked with asterisks.

A

B

Figure S7: Absolute changes in root fresh weight under (A) well-watered and (B) drought stress conditions after inoculation with individual bacterial isolates. The red zero baseline represents the mean of the uninoculated control within each experiment. Positive values indicate an absolute increase, and negative values a decrease in fresh weight compared to the respective control. Individual experiments are separated by dashed lines. Significant plant growth-promotion after inoculation with the isolates compared to the respective control is marked with asterisks.

A

B

Figure S8: Absolute changes in shoot fresh weight under (A) well-watered and (B) drought stress conditions after inoculation with individual bacterial isolates. The red zero baseline represents the mean of the uninoculated control within each experiment. Positive values indicate an absolute increase, and negative values a decrease in fresh weight compared to the respective control. Individual experiments are separated by dashed lines. Significant plant growth-promotion after inoculation with the isolates compared to the respective control is marked with asterisks.

A

B

Figure S9: Absolute changes in shoot length under (A) well-watered and (B) drought stress conditions after inoculation with individual bacterial isolates. The red zero baseline represents the mean of the uninoculated control within each experiment. Positive values indicate an absolute increase, and negative values a decrease in length compared to the respective control. Individual experiments are separated by dashed lines. Significant plant growth-promotion after inoculation with the isolates compared to the respective control is marked with asterisks.

Figure S10: Absolute changes in seedling fresh weight under drought stress conditions after inoculation with individual bacterial isolates. The red zero baseline represents the mean of the uninoculated control within each experiment. Positive values indicate an absolute increase, and negative values a decrease in fresh weight compared to the respective control. Individual experiments are separated by dashed lines. Significant plant growth-promotion after inoculation with the isolates compared to the respective control is marked with asterisks.


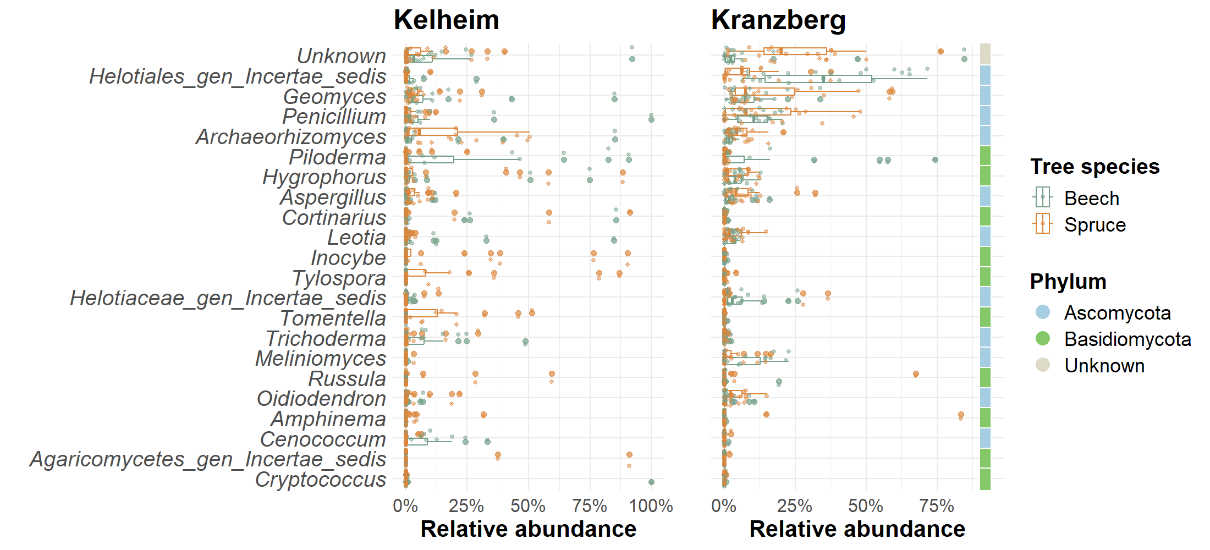

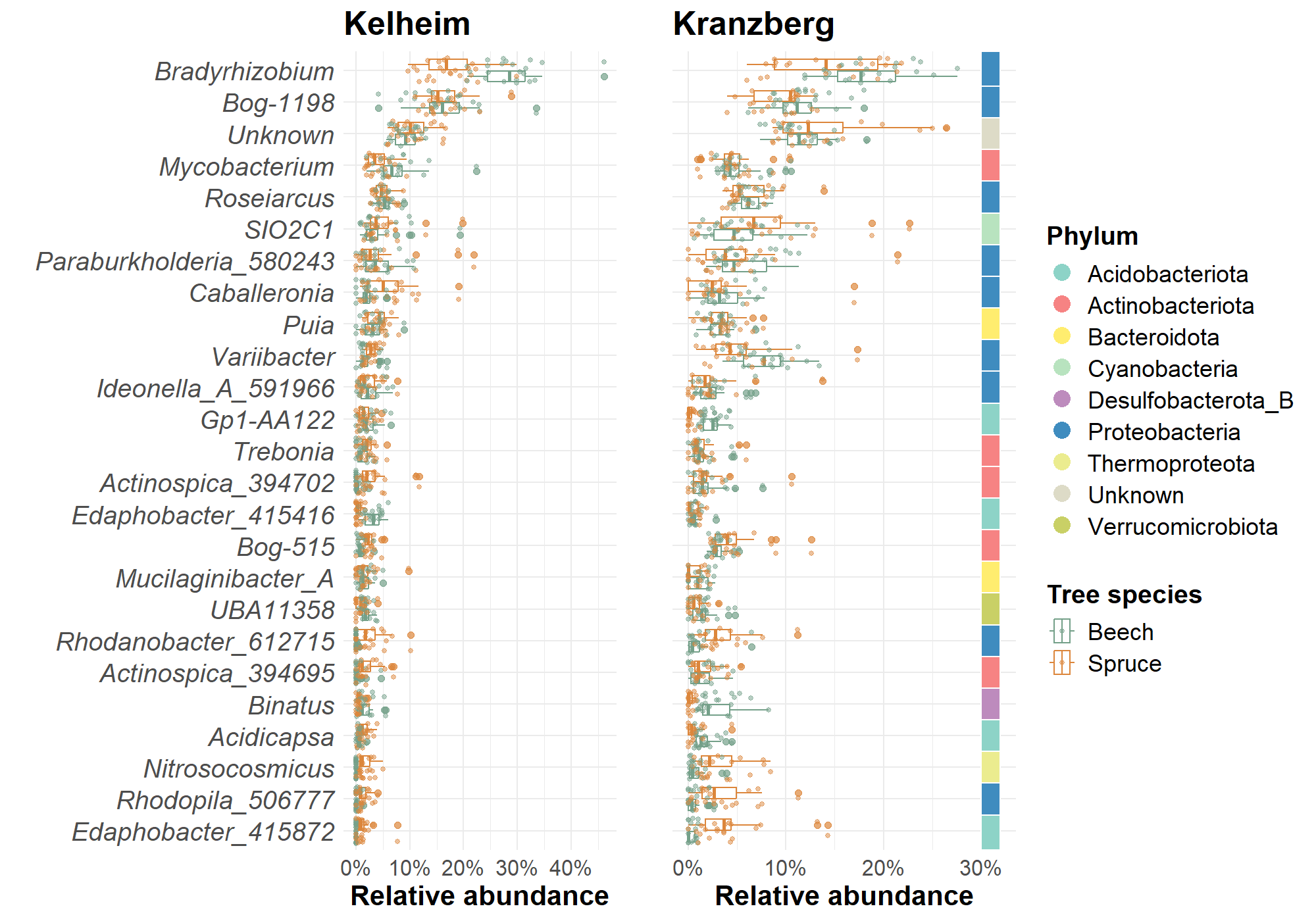


A

B

Figure S11: Relative abundance [%] of the (A) bacterial and (B) fungal genera in beech and spruce rhizosphere communities in Kelheim and Kranzberg, sorted according to their global abundance (descending order). The colors on the right indicate their phylum affiliation.
